# Cognitive benefits of folic acid supplementation during pregnancy track with epigenetic changes at an imprint regulator

**DOI:** 10.1186/s12916-024-03804-2

**Published:** 2024-12-16

**Authors:** L. Hilman, M. Ondičová, A. Caffrey, M. Clements, C. Conway, M. Ward, K. Pentieva, R. E. Irwin, H. McNulty, C. P. Walsh

**Affiliations:** 1 School of Biomedical Sciences, Biomedical Sciences Research Institute, Ulster University, Coleraine, Northern Ireland BT52 1SA UK; 2 Nutrition Innovation Centre for Food and Health (NICHE), School of Biomedical Sciences, Ulster University, Coleraine, Northern Ireland BT52 1SA UK; 3 Department for Cell and Neurobiology, Biomedical and Clinical Sciences Division, Faculty of Medicine, Linköping University, 581 83 Linköping, Sweden; 4 Department of Medical Genetics, Life Sciences Institute, University of British Columbia, Vancouver, BC V6T 1Z4 Canada

**Keywords:** DNA methylation, *ZFP57*, Methylation quantitative trait loci, Linkage disequilibrium, Folic acid

## Abstract

**Background:**

The human *ZFP57* gene is a major regulator of imprinted genes, maintaining DNA methylation marks that distinguish parent-of-origin-specific alleles. DNA methylation of the gene itself has shown sensitivity to environmental stimuli, particularly folate status. However, the role of DNA methylation in *ZFP57*’s own regulation has not been fully investigated.

**Methods:**

We used samples and data from our previously described randomised controlled trial (RCT) in pregnancy called Folic Acid Supplementation in the Second and Third Trimester (FASSTT), including follow-up of the children at age 11. Biometric and blood biochemistry results were examined for mothers and children. Methylation of *ZFP57* was analysed by EPIC arrays, pyrosequencing and clonal analysis, and transcription assessed by PCR-based methods. Functional consequences of altered methylation were examined in cultured cells with mutations or by inhibition of the main DNA methyltransferases. DNA variants were examined using pyrosequencing and Sanger sequencing, with results compared to published studies using bioinformatic approaches. Cognitive outcomes were assessed using the Wechsler Intelligence Scale for Children 4th UK Edition (WISC-IV), with neural activity during language tasks quantified using magnetoencephalography (MEG).

**Results:**

Here we show that methylation at an alternative upstream promoter of *ZFP57* is controlled in part by a quantitative trait locus (QTL). By altering DNA methylation levels, we demonstrate that this in turn controls the expression of the *ZFP57* isoforms. Methylation at this region is also sensitive to folate levels, as we have previously shown in this cohort. Fully methylated alleles were associated with poorer performance in the Symbol Search and Cancellation subtests of WISC-IV in the children at age 11 years. There were also differences in neural activity during language tasks, as measured by MEG. Analysis of published genome-wide studies indicated other SNPs in linkage disequilibrium with the mQTL were also associated with neurodevelopmental outcomes.

**Conclusions:**

While numbers in the current RCT were small and require further validation in larger cohorts, the results nevertheless suggest a molecular mechanism by which maternal folic acid supplementation during pregnancy may help to counteract the effects of folate depletion and positively influence cognitive development in the offspring.

**Supplementary Information:**

The online version contains supplementary material available at 10.1186/s12916-024-03804-2.

## Background

DNA methylation is a key regulator of gene expression and acts as a powerful transcriptional control mechanism in early life and in disease states. Environmental stimuli such as exercise, smoking and nutrition can alter the distribution of DNA methylation throughout the genome, modifying gene transcription and expression [1–3]. This epigenetic mark plays an important role in mammalian development, which is especially apparent at early epigenetic reprogramming phases where the epigenome is erased and reset. During this time, embryonic reprogramming creates an unbiased epigenome, and few genes escape this process.

Imprinted genes are a case of parent-of-origin expression escaping epigenetic reprogramming through maintenance of DNA methylation on one or more imprinting control regions (ICR) [4]. DNA methylation marks are ‘remembered’ and re-established at these genes to allow for normal function and development [5]. These genes have many functions, some of which include regulation of normal embryonal development [6, 7], placental growth [8, 9] and some essential brain functions [10]. The disruption of imprinted gene expression is associated with severe developmental disorders such as Angelman syndrome or Prader–Willi syndrome [11–15]. Specific cellular machinery protects the epigenetically marked ICRs from reprogramming. Most ICRs contain a conserved hexanucleotide sequence TGCmeCGC, associated with the differential methylation. This sequence is recognised by the zinc finger binding protein 57 (ZFP57) [16, 17]. The ZFP57 protein and its cofactor KAP1 recruit the H3K9 methyltransferase SETDB1 and DNA methyltransferase (DNMT) enzymes to maintain histone and DNA methylation marks at ICRs [17, 18].

The structure of the ZFP57 protein in mice is well characterised and consists of a 5’ Krüppel-associated box (KRAB) domain accompanied by 7 C_2_H_2_ zinc fingers (ZF) at the 3’ end [16, 17]. The gene has a maternal–zygotic effect in mice and loss of function in embryonic stem cells (ESCs) results in embryonic lethality [16, 19]. This KRAB zinc finger (KRAB-ZF) protein is part of a large, fast-evolving family that predominantly has a role in retrotransposon repression. However, a subset of proteins has evolved to play an important role in regulating imprinting [20–23], potentially due to similarities between ICRs and retrotransposons [23]. Although a homologue for *Zfp57* exists in humans, the genes and proteins differ significantly in structure and targeting [16, 24]. Mouse *Zfp57* has shown high expression in ESCs and oocytes, but minimal expression is observed in somatic tissues [16]. The human form also demonstrates high ESC expression but in addition is detectable in some somatic tissues [24, 25]. Notably, *ZFP57* expression is only evident at and beyond the blastocyst stage in human tissue [21, 25]. In mice, *Zfp57* is located on chromosome 17 in the major histocompatibility complex (MHC) cluster [26, 27]. In humans, this gene is also located in the MHC class I region on chromosome 6 and has been linked to transient neonatal diabetes mellitus (TNDM) [24, 28]. TNDM patients have shown a pattern of mutations within *ZFP57* that results in a shift in the reading frame or a loss of ion-binding affinity, along with hypomethylation across particular imprinted genes such as *PLAGL1, GRB10* and *PEG3* [28–30]. Additional isoforms have been identified for the human *ZFP57* that are longer than the canonical form and start approximately 3 kilobases (kb) upstream from the transcription start site (TSS) of the original transcript. One of the transcripts contains 4 exons while the other consists of 5 exons [24, 25]. The significance of these extra transcripts is currently unknown, but they start near an extended cytosine–phosphate–guanine (CpG)-rich region which coincides with the differentially methylated regions (DMR) identified by us in an earlier study [31].

We have previously reported that this CpG-rich region at *ZFP57* was one of the top DMR in cord blood from newborns whose mothers had participated in a randomised controlled trial (RCT) of Folic Acid Supplementation during the Second and Third Trimester (FASSTT) of pregnancy [31]. The FASSTT Offspring Trial was conducted to investigate the effects of maternal folic acid (FA) administration on the cognitive development of children, and these were followed up at 3 years, 7 years and 11 years of age [32, 33]. A number of clinically validated cognitive and psychosocial tests, as appropriate for the age of the children at the time, were conducted by trained researchers. For the 3-year-olds, Bayley’s Scale of Infant and Toddler Development, third edition (BSITD-III) was used, which assesses neurocognitive development and exposes possible developmental delays in children less than 42 months old. The Wechsler Preschool and Primary Scale of Intelligence, third edition (WPPSI-III) was used for the 7-year-olds [34] and provides a score for Full Scale IQ as well as for specified cognitive domains (i.e. Performance IQ, Verbal IQ, and Processing speed) [35]. Lastly and of most relevance here, the Wechsler Intelligence Scale for Children fourth edition (WISC-IV) was used for 11-year-olds [36, 37]. It is the most widely used test for evaluating intelligence in older children and is a comprehensive tool providing composite scores for specified cognitive domains -Verbal Comprehension, Perceptual Reasoning, Working Memory and Processing Speed comprising 15 subtests in total -which are combined to give Full Scale IQ [38]. Our previous results showed significantly higher scores in Verbal Comprehension (girls only), and in two Processing Speed tests in the children of mothers assigned to FA supplementation, compared to placebo, during pregnancy [33]. As a second, independent measure of differences in cognitive development, magnetoencephalographic (MEG) brain imaging was also used in a subgroup of the children, assessed at 11 years of age. This is a non-invasive technique analysing the magnetic fields that accompany specific neuronal currents produced by brain activity and provides a quantitative physical measurement of difference in neuronal activity [39]. The results showed increased power in the beta (13–30 Hz) and high gamma (49–70 Hz) bands in neuronal responses to language tasks in the children of the FA-supplemented mothers, indicating more efficient semantic processing of language compared to placebo [32, 33].

In light of the important role of *ZFP57* in regulating imprinted genes, which often have neurodevelopmental effects, as well as the colocalization of methylation differences with the upstream promoter, we have further investigated this region in our maternal–offspring cohort to look for associations between the methylation status and cognitive outcomes. We have identified additional isoforms in multiple cell lines that originate within this DMR. We discovered a single-nucleotide polymorphism (SNP) within the *ZFP57* promoter/DMR that dictates methylation of the surrounding region. This SNP plays a role in regulating transcription from the upstream TSS. A CCCTC-binding factor (CTCF) binding site also overlaps this region and may influence expression. Lastly, we identified a relationship between the methylation quantitative trait locus (mQTL) and cognitive development in early life (for a graphical abstract see Additional File 1: Fig. S1).

## Methods

### Study design

Umbilical cord blood samples were acquired from the FASSTT trial cohort (ISRCTN19917787), fully described elsewhere [40, 41]. Briefly, women with singleton pregnancies who had taken FA at 400 µg per day during the first trimester (as recommended in the UK and worldwide for prevention of neural tube defects, NTD) were recruited to this double-blinded RCT at the 14th gestational week from antenatal clinics at the Causeway Hospital, Coleraine, Northern Ireland. Individuals were excluded if they were taking medication known to interfere with FA or other B vitamin metabolism or if they had any vascular, renal or gastrointestinal disease, epilepsy or had a previous NTD-affected pregnancy. Eligible participants were randomised at the end of their first trimester to receive either 400 µg/day of FA (*n* = 96) or placebo (*n* = 94) until the end of pregnancy. Umbilical cord blood samples were collected at delivery. The trial was completed in full by a total of 119 women. Of these, DNA was available for extraction from 93 cord blood samples (52 placebo and 41 FA-supplemented). Notable biometric measures included birth weight, length, head circumference, mode of delivery and Apgar score. Additionally, blood samples were analysed for serum folate, red blood cell (RBC) folate, serum B12 and plasma homocysteine in maternal and cord blood.

At 11 years of age, a total of 68 children were followed up for assessment of cognitive performance along with general health characteristics [32]. For the current report, those with matching cord blood samples collected at birth were used in the current epigenetic analysis (*n* = 67). A subsample of these children (*n* = 23) was also willing to provide a blood sample (13 from placebo and 10 from FA-supplemented mothers). The samples were analysed for red blood cell folate, serum B12 and plasma homocysteine concentrations.

### Cognitive assessment

Cognitive assessments were administered at ages 3, 7 and 11 years as described in detail elsewhere [32, 33]. Of most relevance here, the Wechsler Intelligence Scale for Children 4th UK Edition (WISC-IV) test was used at age 11 to assess cognitive function using Q-interactive™ software (Pearson Education Ltd., UK), with the tests conducted on iPads [42]. The WISC-IV, which is appropriate for children ages 6 years to 16 years and 11 months, encompasses a range of tests including Verbal Comprehension, Perceptual Reasoning, Working Memory and Processing Speed (which included subtests for symbol search and cancellation), along with providing a combined full-scale IQ score representing general intelligence of the child. This was conducted on 68 children at age 11 from the FASSTT study [32]. All tests were conducted in a single session lasting between 90 and 120 min per child. The assessment room was bright, well-ventilated and free from distractions or interruptions to ensure the ideal testing environment. The same trained researcher performed each assessment on all children and was blinded prior to assessment of the child’s FASSTT study intervention group (placebo or FA-supplemented).

### Brain imaging analysis

In addition to the clinical psychological assessment, a subgroup of the children also completed a MEG brain imaging (*n* = 33) assessment at the Northern Ireland Functional Brain Mapping (NIFBM) facility in the Intelligent Systems Research Centre, Ulster University. Prior to conducting the MEG assessment, the children watched a child-friendly introductory video to explain MEG, produced by Aston University [43]. Children were provided with earphones and instructed to remain as still as possible in the MEG machine. Bipolar electrodes tracked eye movements and heartbeat rhythms, and head movement was recorded through a head–position indicator with coils placed on the child’s scalp. Head shape and head–position indicator coil position were defined using a 3D digitiser (Fastrak, USA). The head–position indicator digitisation points were realigned to the individual head shape for each participant and then registered to the recorded MEG data to allow for anatomically informed MEG analysis. Taking into consideration the previous analysis at 7 years that showed FA-supplemented participants from the FASSTT study scored higher in verbal IQ [33] and observational data correlating maternal folate deficiency in pregnancy with lower language abilities in the children [44], a language task was designed to assess the underlying neurophysiological basis of language processing for the age 11 years assessment using MEG. A high-level semantic language task [45] was adapted for this task to compare differences in neural responses to two sentences. These were described as either congruent or incongruent. For example, ‘The baby was thirsty and wanted a drink of milk/fire’, where ‘fire’ or ‘milk’ renders the sentence semantically incongruent or congruent, respectively. The Elekta Neuromag 306-channel MEG system was used to record MEG responses. In response to the language paradigm, band power scores were recorded for 116 brain regions and averaged to six standard spectral bands. These included big band (1–48 Hz), theta (4–8 Hz), mu (8–12 Hz), beta (13–30 Hz), low gamma (30–48 Hz) and high gamma (49–70 Hz).

### DNA extraction and bisulfite conversion

The umbilical cord blood samples were collected and stored in − 80 °C until required. DNA was extracted from blood samples by using QiAMP DNA Blood Mini kit (Qiagen), according to the manufacturer’s instructions. DNA was assessed for quality control by agarose gel electrophoresis and using the Nanodrop spectrophotometer to assess purity and yield (Labtech International, Ringmer, UK); 500 ng of genomic DNA from cell pellets was bisulfite converted in house using the EZ DNA Methylation Kit (Zymo Research, California, USA) when used for pyrosequencing.

### Polymerase chain reactions and DNA methylation analysis

DNA and/or bisulfite-converted DNA was used for PCR reactions to amplify regions for multiple purposes. The list of primers used in this study is indicated in Additional File 1: Table S1. For pyrosequencing reactions, one primer was labelled with 5’ biotin tag (Metabion, Munich, Germany) and used to amplify the untreated DNA (used for SNP/AQ assay) or bisulfite-converted DNA (used for pyrosequencing). A standard PCR reaction (used for both assays) was run with conditions: initial denaturation at 95 °C for 15 min, followed by 45 cycles of 95 °C for 30 s, 56 °C for 30 s and 72 °C for 30 s, with final elongation at 72 °C for 10 min. The PCR products were verified on an 1.5% agarose gel. To amplify regions of interest to assess DNA methylation and for the SNP genotyping, primers covering regions of interest were designed using PyroMark Assay Design Software 2.0 (Qiagen).

A pyroassay was designed using the PyroMark Q48 Autoprep software (Qiagen) to analyse the DNA methylation percentage for each CpG site covered by the primers and an in-assay bisulfite conversion technical control was incorporated according to the manufacturer’s recommendations to ensure efficient conversion of all CpG sites. For the SNP pyroassay, a separate assay was designed using the same software. This assay quantified the nucleotide base present at each SNP investigated.

### Cell culture

The human colorectal cancer cell line, HCT116, as well as the *DNMT1* and *DNMT3B* double knockout (DKO) cells [46] were cultured in 1 g/L glucose Dulbecco’s modification of Eagle’s medium (DMEM) supplemented with 10% foetal bovine serum (FBS) and 1 × NEAA (all Thermo Fisher Scientific, Loughborough, UK). The human neuroblastoma cell line (SH-SY5Y) was seeded onto a 90-mm plate and cultured in DMEM/F12 medium supplemented with 10% FBS (Thermo Fisher Scientific, Loughborough, UK). The following day SH-SY5Y cells were treated with medium containing 5′aza-2-deoxycytidine (5-aza-dC) (Sigma-Aldrich, Dorset, UK) at a final concentration of 1 μM. Treatment was renewed at 24-h intervals up to 72 h. Cells were then harvested for DNA and RNA extraction. All cells were incubated at 37 °C and in 5% CO_2_.

### RNA extraction and cDNA synthesis

RNA was extracted from cell pellets using the RNeasy Mini Kit (Qiagen, Crawley, UK) according to the manufacturer’s instructions. The quantity and quality of RNA was verified by gel electrophoresis and with Nanodrop 2000 spectrophotometer (Labtech International, Ringmer, UK) ultraviolet (UV) absorbance readings at 260/280 and 260/230 nm; 500 ng of RNA was used for complementary DNA (cDNA) synthesis with 250 ng random primers, 1X U buffer RT and 200 U RevertAid reverse transcriptase (RT) (all Thermo Fisher Scientific, Loughborough, UK). The cDNA synthesis reaction was carried out under the following conditions: 25 °C for 10 min, 42.5 °C for 50 min and 70 °C for 10 min.

### RNA-Seq analysis

A human placental dataset, GSE56781 [47], was used to assess transcript variants of the *ZFP57* gene using *GEOquery* (2.46.15). The processing of the RNA sequencing (RNA-Seq) data was performed on the usegalaxy platform (usegalaxy.com). The reads were mapped with the *TopHat2* package and visualised via Integrative genomics viewer [48], which was used for Shashimi plot visualisation for splice variants. The data were normalised using *Deseq2* and the *HTSeq/DESeq2* pipelines, available on usegalaxy.com.

### Bioinformatic analysis

Initial genome-wide screening for methylation differences in the FASSTT cord blood samples using EPIC arrays has been previously described [31, 49]. In brief, *GenomeStudio* (Illumina v3.2) was used for initial data processing and imported into the *RnBeads* package (version 1.6.1) [50] in the freely available RStudio platform (version 3.1.3). Samples were quality controlled and normalised and a *limma*-based linear model was used. Methylation was represented as *β* values (0 was unmethylated, 1 was fully methylated) and plotted against genomic loci based on Human Genome Build 19 (*hg19)* using CandiMeth workflow on *GALAXY* software [51] for visualisation on the University of California at Santa Cruz genome browser (https://genome.ucsc.edu/) [52].

#### Linkage disequilibrium analysis

Online software using the published database from the National Institutes of Health was used to calculate linkage disequilibrium (LD) values between each SNP and mQTL discussed [53]. *LDtrait* is an online tool used to identify LD associations to published phenotypes in the form of *R*^2^ or *D*’ values ranging from 0 (non-LD) to 1 (complete LD) [54]. The heatmap was generated using *LDmatrix*, which is an online tool designed to create an interactive heatmap matrix of pairwise LD statistics (LDmatrix (nih.gov)). The Functional OR Genomic Element Database (FORGEdb) was included in the LDmatrix software and calculates a score out of 10 for how likely a genetic variant is to be a regulatory variant.

#### Statistical analysis

Statistical analysis was performed using the Statistical Package for the Social Sciences (SPSS) software (version 29.0.1.0; SPSS UK Ltd., Chertsey, UK). The results are expressed as mean ± SD, except where otherwise stated. Differences between treatment groups for participant characteristics were assessed using an independent *t*-test for continuous variables or chi-square for categorical variables. A Student *t*-test was conducted for measuring differences in mean DNA methylation between different cell lines (HCT116, DKO, SH-SY5Y, Aza-treated). Pyrosequencing data were analysed using a paired samples *t*-test to identify statistical differences between pre-intervention and post-intervention groups. Regression and non-parametric Spearman’s rank correlation analyses were conducted in SPSS for methylation comparisons between cord blood and blood at 11 years of age. Genotype and methylation comparisons for mQTLs (rs365052 and rs2747429) were conducted using ANOVA. Analysis of scaled processing speed differences between rs365052 genotype and FASSTT intervention groups were conducted using 2-way ANOVA while 2-way ANCOVA controlling for sex was also conducted for MEG comparisons. A *p* value < 0.05 was considered significant in all tests.

## Results

### Overview of the FASSTT cohort and their offspring at 11 years

**Fig. 1:**
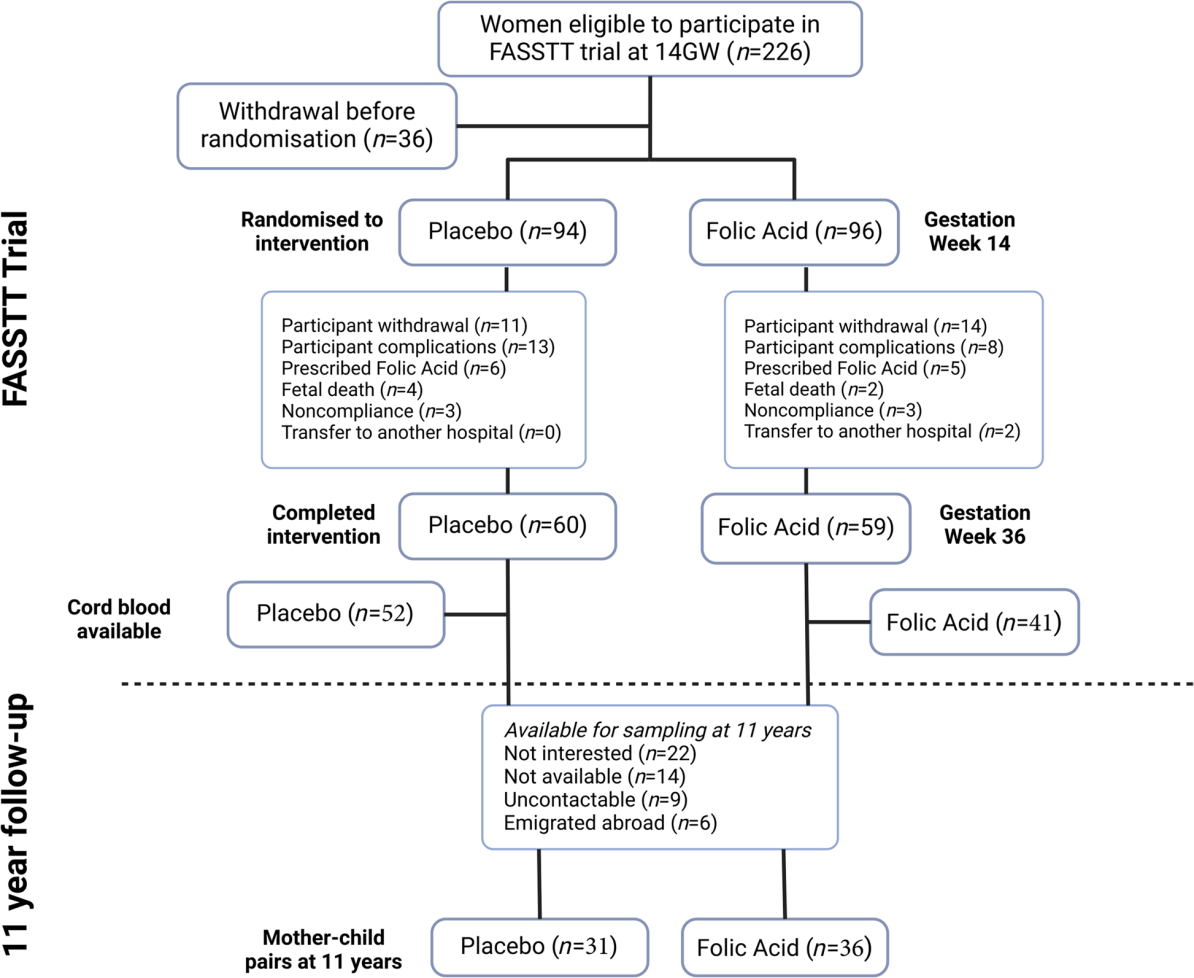
Outline of the FASSTT randomised controlled trial and 11-year follow-up study. Eligible women during the first trimester of pregnancy (*n* = 226) were offered a place on the trial. Some withdrew (*n* = 36) while the remaining were randomised into two groups: placebo (*n* = 94) and folic acid (*n* = 96). Withdrawal after randomisation (*n* = 25) or reasons for exclusion from the intervention are indicated. A total of 119 women completed the trial (placebo, *n* = 60; folic acid, *n* = 59). Blood samples were taken at gestational weeks (GW) 14 (pre-intervention) and 36 (post-intervention). A total of 93 cord blood samples (placebo, *n* = 52; folic acid, *n* = 41) were available for DNA analysis. A follow-up study carried out cognitive assessments on the same children at age 11 years: 67 had both cognitive assessments and DNA samples available from cord blood (placebo, *n* = 31; folic acid, *n* = 36)

The current analysis investigates a subgroup of previously assessed samples from the FASSTT study for which cord blood was available, as outlined in Fig. 1. A summary of the characteristics and biochemical measures for these children are detailed in Table 1. There were no significant differences between the placebo and the FA-supplemented groups for any neonatal characteristics including gestational age, sex, birth weight, length, head circumference, Apgar scores at 5 min or birth by caesarean section. Corresponding B vitamin biomarkers were assessed in both cord blood (*n* = 93) and at 11 years of age (*n* = 23). Not all the children at age 11 that returned for body measurements consented to blood draws and those used in this analysis had to have a matching cord blood sample. B vitamin biomarkers in the cord blood indicated significantly higher concentrations in the FA group compared with placebo for serum folate (*p* < 0.001) and red blood cell folate (*p* = 0.003), whereas plasma homocysteine and vitamin B12 concentrations in cord blood did not differ between the groups. At age 11 years, there were no differences in B vitamin biomarker concentrations between the treatment groups for RBC folate, serum B12 or plasma homocysteine (*p* = 0.449).

**Table 1:** General characteristics for the FASSTT participants in this study

Characteristics	Placebo			Folic acid			*P value*
	*N*	Mean	SD	*N*	Mean	SD	
Neonatal characteristics	*n* = 52			*n* = 41			
Gestational age (weeks)		40.13	1.19		39.98	1.11	0.511
Sex, male *n* (%)		26 (50)	-		22 (53.7)	-	0.726
Birth weight (g)		3555.75	486.33		3557.07	464.62	0.989
Birth length (cm)		51.28	2.60		51.12	2.17	0.763
Head circumference (cm)		35.67	1.26		34.82	1.37	0.563
Apgar score at 5 min		8.94	0.37		9.02	0.27	0.234
Caesarean *n* (%)		12 (23.1)	-		10 (24.4)	-	0.882
B vitamin biomarkers (cord blood)							
Serum folate (nmol/L)		66.80	24.39		91.66	36.71	< 0.001*
RBC folate (nmol/L)		1437.76	584.06		1877.33	700.93	0.003*
Serum B12 (pmol/L)		277.29	154.66		251.39	107.07	0.369
Plasma homocysteine (µmol/L)		11.19	6.85		11.11	4.87	0.952
Characteristics at 11 years of age	*n* = 31			*n* = 36			
Age (years)		10.78	0.12		10.82	0.43	0.586
Sex, male *n* (%)		15 (48.3)	-		15 (41.6)	-	0.317
Weight (kg)		39.31	9.62		37.68	7.15	0.430
Height (cm)		147.06	6.18		147.63	8.15	0.810
BMI (kg/m2)		18.03	3.66		17.48	2.80	0.487
Head circumference (cm)		55.18	2.79		54.69	2.15	0.427
B vitamin biomarkers (11 years of age)	n = 13			n = 10			
RBC folate (nmol/L)		581.98	144.24		616.94	291.48	0.709
Serum B12 (pmol/L)		511.76	186.18		522.40	134.80	0.880
Plasma Homocysteine (µmol/L)		7.02	1.39		7.60	2.18	0.449

Statistical comparisons by independent *t*-test (continuous variables) or chi-squared test (categorical variables) *BMI* body mass index, *RBC* red blood cell, *SD* standard deviation, *N* number of samples

^*^p < 0.05

### Methylation of the ZFP57 promoter is influenced by both folic acid and haplotype

**Fig. 2:**
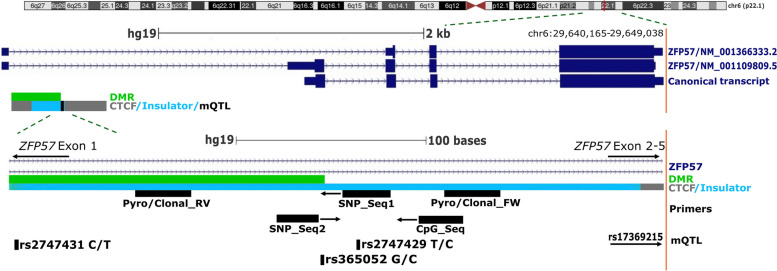
Schematic overview of ZFP57 isoforms and regions used for analysis in this study. Top, overview of human chromosome 6 (genome release hg19), with ZFP57 alternative isoforms (dark blue) starting at a differentially methylated region (green) and a CTCF binding site (grey) with insulator activity (light blue). The mQTLs of interest is highlighted in black. Bottom, a zoomed-in section of the differentially methylated region (green) within the first ZFP57 intron with primers used for DNA methylation (CpG pyroassay) and genotype (SNP pyroassay) analysis. Informative SNP variants from this study are shown, the mQTLs are shown in black and some of the other SNPs in the region also shown. The CTCF binding site (grey) and insulator (light blue) are shown in comparison to primer and SNP locations

Our epigenome-wide analysis using the EPIC array previously highlighted a DMR approximately 3 kb upstream of the canonical start site of the imprint regulator *ZFP57* as the top-scoring region on RnBeads [31]. This score is calculated through a combination of change in mean methylation, the quotient of the mean methylation and the combined *p-*value. The resultant score ranked regions based on the changes in DNA methylation altered by FA supplementation in cord blood samples from the FASSTT cohort. A schematic overview of the *ZFP57* locus is shown in Fig. 2, highlighting the position of the DMR as well as the canonical transcript. The DMR covers 15 probes on the EPIC array (Additional File 1: Table S2) and showed an average gain in methylation of 6.23% in the FA-supplemented samples, *p* = 0.012 (Table 2). We confirmed a similar, though non-significant, mean difference in methylation (5.14%, *p* = 0.169) at 6 CpG overlapping the DMR using a CpG pyroassay (Table 2), which is a more gene-specific and scalable assay. We then used the same CpG pyroassay to examine methylation at this region in the children from the FASSTT offspring cohort at 11 years. This showed that while there was still a gain in methylation in the FA-supplemented group, the difference was much smaller (2.04%, *p* = 0.601), confirming that the effect of FA supplementation was greatest at the end of pregnancy.

**Table 2:** Epigenetic characteristics in cord blood and blood from children at age 11

		Placebo			Folic acid				*p* value
Characteristics	No. CpG	*N*	Mean	SD	*N*	Mean	SD	Change in methylation (%)	
*ZFP57* DMR methylation (Cord blood)	6	52	61.22	18.51	41	66.36	16.78	5.14	0.169
ZFP57 DMR methylation (11-year blood)	6	13	54.53	9.48	10	56.57	8.64	2.04	0.601
ZFP57 DMR methylation (EPIC 850 k array)	15	45	64.38	14.63	41	70.61	12.22	6.23	0.012

Statistical comparisons by independent *t-test* (cord blood and 11-year blood) and using *Limma* to generate comb-*p* value for EPIC array. DMR methylation performed by pyrosequencing of 6 CpG sites (cord blood and 11-year blood) and by EPIC array

*SD* standard deviation, *N* number of samples

^*^p < 0.05

Previous reports have indicated that SNPs at *ZFP57* may also drive DNA methylation. Using data from the 1000 Genomes Project, we identified two common SNPs within the upstream region: rs365052 (G/C 66:33%) and rs2747429 (C/T 27:73%). We first designed a SNP pyroassay to assess polymorphism frequency at rs365052 in our cohort (Fig. 2, SNP Seq1). This confirmed that FASSTT participants showed variation in allele types at this SNP, with evidence for all three genotypes being present, C/C being least frequent. Analysis by Fisher’s exact test indicated no overall difference in allele frequency between the FA-supplemented and placebo groups (*p* = 0.524, Additional File 2: Table S3). We also typed a subset of samples for rs2747429, which has been described previously [55], using a SNP pyroassay (SNP Seq2) and found similar results (*p* = 0.819, Fisher’s exact test, Additional File 2: Table S4). Results from chi-squared tests were also non-significant.

**Fig. 3:**
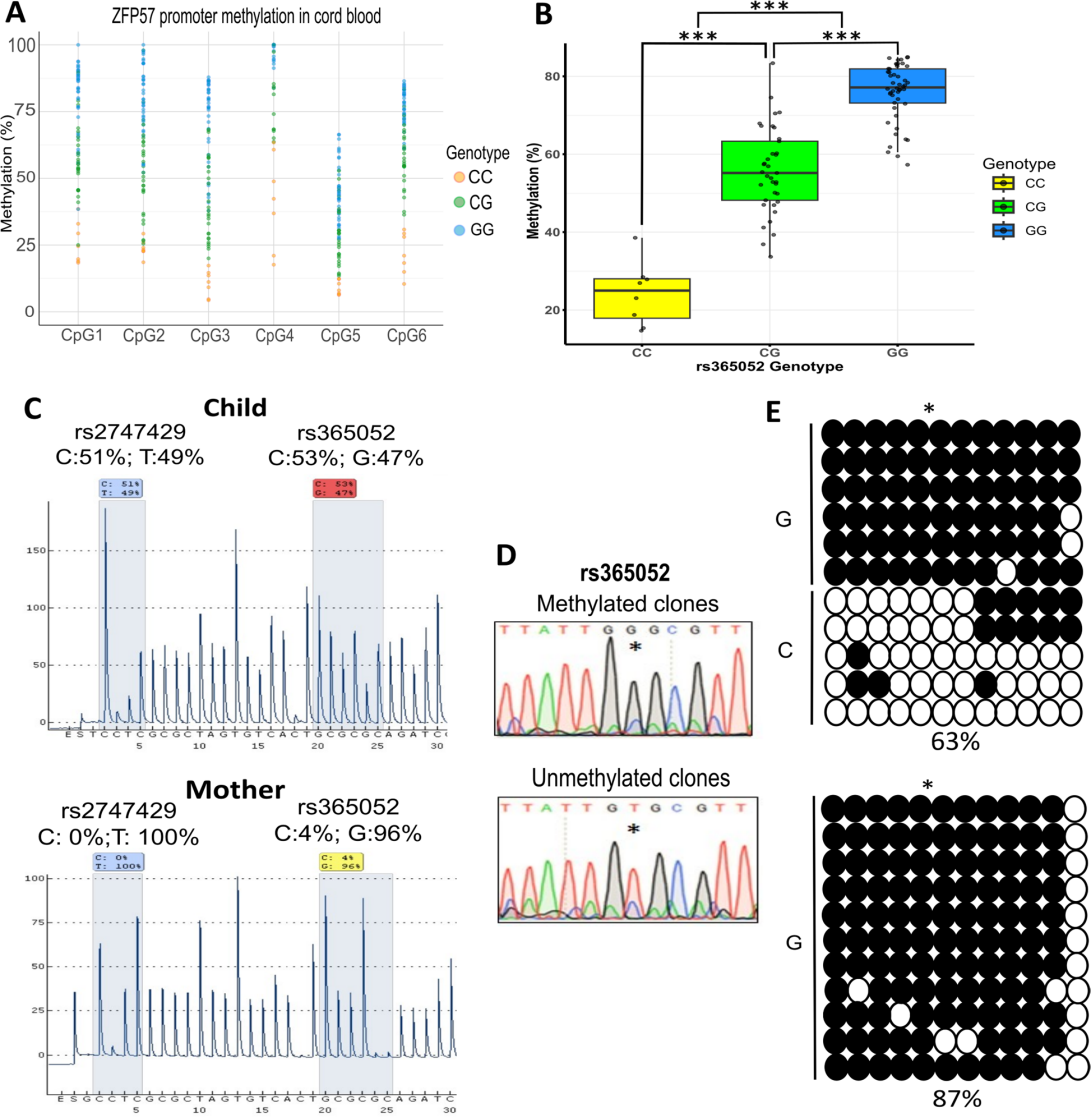
Methylation quantitative trait loci in the differentially methylated region of ZFP57.** A** Detailed CpG methylation analysis of the ZFP57 alternative promoter analysed by pyrosequencing in the cord blood. DNA methylation of individual CpG sites is shown for CpG sites 1–6, using primers in Additional File 1: Table S1 (CpG pyroassay). Yellow dots represent samples with C/C genotype within the rs365052 SNP, green represent samples with C/G and blue with a G/G SNP genotype.** B** Boxplot with jitter data of ZFP57 methylation by the rs365052 SNP demonstrating significant methylation differences between different genotype groups in blood. Genotype analysis performed by allele quantification (AQ). Statistically significant values are marked as: *** indicates *p* < 0.001, conducted by ANOVA. **C** Identification of heterozygous gDNA sample (child) for the rs365052 (C/G) region assessed by the AQ assay (SNP pyroassay 1). Allele frequency of 2 SNP variants rs2747429 (C/T) and rs365052 (C/G) indicated by the common SNPs analysis. Percentage of the allele frequency is highlighted in boxes with colour identification based on the quality of the run (blue—very good, yellow—satisfactory, red—poor quality). AQ assay of matching homozygous maternal gDNA within the SNP variant rs365052 (G/G) extracted form blood.** D** Sanger sequencing analysis identifying mQTL within the rs365052 SNP that in methylated and unmethylated clones, SNP shown at asterisk.** E** Bisulfite sequencing clonal analysis of the region from Fig. 2 for child (C/G) and mother (G/G), showing methylation distribution in individual clones. Polymorphic base located between the fifth and sixth CpG site (asterisk) corresponds with the rs365052. Full circle represents methylated and empty unmethylated CpG sites

To assess if genotype at the rs365052 variant is linked to DNA methylation within the region we analysed 6 CpG sites around the SNP (Fig. 3A). DNA methylation across all CpG sites were varied in our samples, ranging from 4.33% to 100% with an average methylation of 63.25% (SD = 26.24). Figure 3A shows methylation levels at all 6 CpG sites assayed (*n* = 93), colour-coded by genotype (homozygous C/C yellow; heterozygous C/G green and homozygous G/G blue). While methylation varies by site, with CpG4 showing highest levels overall, and CpG5 lowest, it can be seen that the C/C homozygotes tend to have methylation at the lower end of the range, while G/G are at the high end (Fig. 3A). This can be more clearly seen when methylation is averaged by genotype (Fig. 3B and Table 3). The C/C genotype had low levels of methylation (mean = 24.21, SD = 7.97), the heterozygous C/G an intermediate level of methylation (mean = 55.79, SD = 10.91) and the homozygous G/G showed the highest methylation levels (mean = 75.97, SD = 7.41). Standard deviations were tightly clustered and the methylation differences between each genotype were statistically significant by ANOVA analysis (*p* < 0.001, Table 3). These data strongly suggest that this SNP acts as a mQTL in this cohort.

**Table 3:** Average methylation levels relevant to the SNP variant rs365052

rs365052 variants	Av. Meth	SD	Min	Max	*N*	*p* value
CC	24.21	7.97	14.69	38.54	8	*CG* = *5.10E-9*
						*GG* = *5.10E-9*
CG	55.79	10.91	33.67	83.37	37	
GG	75.97	7.41	57.29	84.96	48	*CC* = *5.10E-9*
						*CG* = *5.10E-9*

*Av. Meth.* average methylation between 6 CpG sites measured by pyrosequencing, *SD* standard deviation, *min* minimum, *max* maximum, *N* number of samples, significance indicated by *p* < 0.05 and is measured by ANOVA between the genotypes

We also confirmed these findings using the previously reported SNP rs2747429, which is 21 base pairs downstream of rs365052. A similar allele quantification pyrosequencing technique (SNP Seq2, Fig. 2) was performed on non-bisulfite-treated DNA to determine the SNP genotypes present. Since this has been examined before, we only looked at a subset of 22 cord blood samples for this additional SNP (rs2747429) and cross compared to the local DNA methylation analysis already performed for the first SNP analysed (rs365052). Both mQTLs are very close together and are inherited together and 18/22 of these samples confirmed this through SNP pyrosequencing in our cohort. A clear relationship between methylation and genotype was also evident for rs2747429 (Additional File 2: Table S5 and Fig. S2). The C/C genotype (*n* = 3) corresponded with a low mean methylation (18.99%) like rs365052. The C/T heterozygous genotype (*n* = 4) showed an intermediate level of mean methylation (48.28%), and the homozygous T/T genotype (*n* = 15) displayed a high level of mean methylation (72.24%). The standard deviations were low and tightly clustered between groups, C/C (SD = 6.88), C/T (SD = 4.08) and T/T (SD = 8.60) (Additional File 2: Table S5). Significant differences in methylation could be seen between the three genotypes for rs2747429, measured via ANOVA (*p* < 0.001). This demonstrated a clear mQTL pattern in rs2747429, similar to rs365052. Pyrosequencing assays which covered both SNPs suggest that the C allele at rs365052 is in phase with the C at rs2747429, with the G allele at the former in phase with T allele at the latter (Fig. 3C).

While these data strongly suggested that the SNPs were acting as mQTLs, average methylation levels in homozygotes in cord blood did not reach 100% or 0% and neither pyrosequencing nor the array can absolutely determine allele phasing. It was therefore unclear whether methylation would vary stochastically on each haplotype, and we could not fully rule out other effects such as parental imprinting. To surmount this difficulty, we designed primers to allow cloning and sequencing of the bisulfite-converted DNA. The region cloned included the rs365052 SNP allowing simultaneous determination of haplotype and methylation across the region analysed (Fig. 3D). Figure 3E shows the results from the mother and child pair from Fig. 3C. The clonal analysis results shown in Fig. 3E represent the methylation distribution of the CpG sites around rs365052. Each row represents a separate clone, while each circle indicates an individual CpG site. An empty circle represents an unmethylated CpG site and a filled circle represents a methylated CpG site. The mQTL is location between CpG’s five and six as shown by the asterisk. In the child who was identified as heterozygous (C/G) for rs365052 in Fig. 3C, a clear pattern of allele specific methylation is shown. Almost all of the clones that contained a C allele at rs365052 show either no methylation or very little methylation within this region. The opposite can be seen for the clones displaying a G at the mQTL. These clones collectively are almost fully methylated. The total percentage methylation across all clones is 63%, which is in a reasonable range for this heterozygous mQTL genotype. In contrast, the mother of this pair contained a different genotype. A G/G homozygous genotype is present here and shows very high levels of in *cis* methylation in every clone. A total methylation percentage equates to 87% in this sample combining all clones, a strong indicator that the G allele is the driver of methylation for this mQTL. These results show that the SNP at rs365052 (and by extension rs2747429) act as mQTLs at this locus, with the presence of a G at rs365052 and a T at rs2747429 being indicative of a fully methylated allele, while Cs at both SNPs are a hallmark of an unmethylated allele, with heterozygotes having intermediate levels of methylation, typical of mQTL loci.

Having established that both SNPs act as mQTLs, we examined samples from the children at 11 years to determine if the levels of methylation associated with each haplotype were similar. Whole blood DNA was available from 23 participants at this timepoint and was extracted and assayed using both the rs365052 SNP and CpG pyroassays. Table 4 summarizes the results of these analyses. Although there was only one C/C homozygote present, the C/G and G/G genotypes were closer in number. While there were still significant differences in average methylation between each genotype (*p* = 0.012) at this timepoint, the difference was smaller than in the cord blood at birth (*p* < 0.001). This was largely due to lower average methylation in the G/G homozygotes (55% at 11 years of age compared to 75% at birth) rather than changes in the heterozygotes (50% at 11 years of age compared to 55%, respectively). Correlation analysis highlighted that a moderate positive correlation (correlation coefficient, 0.423; *p* = 0.044) can be seen between the two age timepoints for blood methylation (Additional File 2: Table S6 and Fig. S3). This indicates that the methylation at the rs365052 mQTL at birth and 11 years later are still comparable, following the same trend, but with reduced mQTL effects at 11 years of age.

**Table 4:** Average methylation levels relevant to the rs365052 SNP at 11 years old

rs365052	Av. Meth	SD	Min	Max	N	*p* value
CC	35.17	-	35.17	35.17	1	*0.012*
CG	52.63	7.73	40.83	62.33	9	
GG	58.90	7.40	45.17	72.83	13	

*Av. Meth.* average methylation within 6 CpG sites measured by pyrosequencing, *SD* standard deviation, *min* minimum, *max* maximum, *N* number of blood samples; significance indicated by *p* < 0.05 and measured by ANOVA

### DNA methylation of the ZFP57 promoter controls expression of the alternative ZFP57 isoforms

**Fig. 4:**
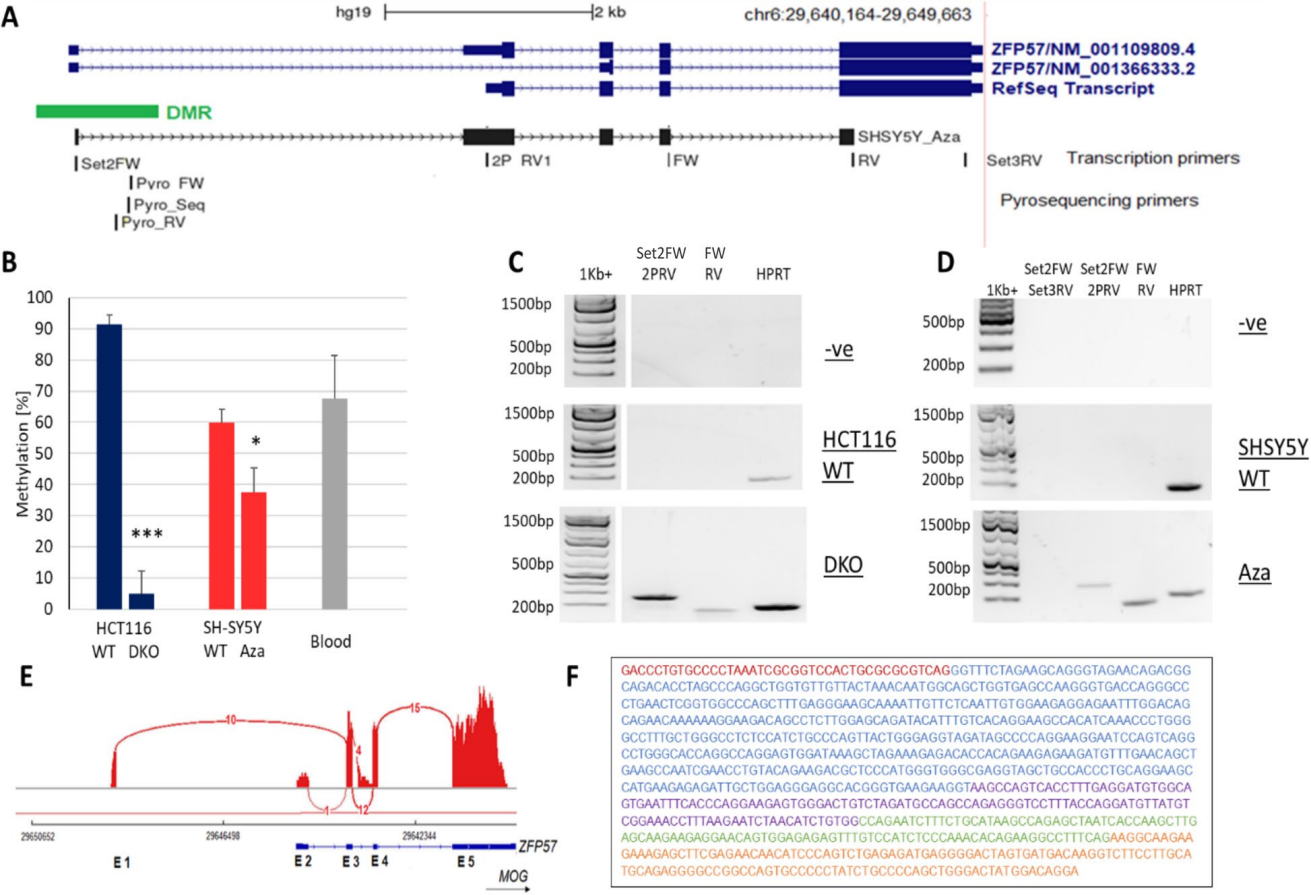
Alternative promoter variant of the imprinted gene regulator ZFP57 is regulated by DNA methylation***.* A** University of California, Santa Cruz (UCSC) browser screenshot of different *ZFP57* isoforms (blue) and of the isoform sequenced in this study in SH-SY5Y demethylated cells (black). Transcription primers and primers used for methylation analysis pyrosequencing are showed. **B** Methylation response of the DMR/promoter region to various demethylation techniques in different cell lines. Human colorectal cancer cell line wild-type (WT) HCT116 and DKO (blue), neuroblastoma cell line SH-SY5Y and same cell line treated with demethylating agent 5-deoxyazacytidine (red), and blood (grey) methylation used as a control. Statistically significant values are marked as: single asterisk indicates *p* < 0.05, triple asterisks indicate *p* < 0.001 conducted by student *t-*test. **C** RT-PCR showing upregulation of the individual exon junctions using the primers indicated in **A** in human colorectal cancer cells, **D** neuroblastoma cell line SH-SY5Y. Alternative exon and all transcripts marked, raw images found in Additional file 4: Fig. S5. 1 kb plus ladder used, -ve, negative control (no template control); HPRT loading control. **E** Shashimi plot, alternative spliced variants of *ZFP57* in placenta confirming alternative promoter usage within the region upstream the original *ZFP57* RefSeq transcript (blue). Spliced junctions represented as red bars; arcs display the number of reads split across the junction. Exons of the alternative isoform are showed below (E1–E5). Genomic coordinates and the gene annotation track are shown below the junction tracks and the downstream *MOG* gene is indicated in black. **F** Isoform detected in demethylated SH-SY5Y. cDNA sequence of *ZFP57* gene with individual exons marked in different colours

We next investigated the *ZFP57* isoforms to determine if DNA methylation at the DMR had any control over their expression. The primers and sequences examined are shown in Fig. 4A. Cell line pairs with opposite methylation patterns were used. The first pair was the human colorectal cancer cell line (HCT116) and its double knock out (DKO) daughter cell line. The DKO contained mutations at *DNMT3B* and *DNMT1* resulting in very low methylation presence. The second pair was the neuroblastoma cell line (SH-SY5Y) and its 5-deoxy-aza-cytidine (Aza)-treated counterpart. Aza treatment resulted in significant demethylation of the genome including the DMR region of interest. Similar to prior experiments, pyrosequencing determined the methylation presence surrounding the mQTL across these cell lines (Fig. 4B). The DNMT knockouts in the HCT116 cells resulted in nearly complete demethylation of the region. A reduction from 90% to less than 10% (blue bars, *p* = 0.00017). The neuroblastoma cell line represented a tissue that expresses *ZFP57* at higher quantities, providing more biologically relevant results. In untreated cells, the average methylation was 60% which decreased to below 40% after Aza treatment (red bars, *p* = 0.014). Blood DNA methylation was used as a control comparison (grey bar). It was evident that DNA methylation at the DMR could be reduced significantly in multiple cell lines using these experiments.

RT-PCR primers were designed to determine the effect reducing DNA methylation had on the upstream promoter’s abilities. Exons 1 and 2 were amplified in the first set to detect the alternative isoforms in the HCT116 wild type, DKO, SH-SH5Y and Aza-treated cell lines (Fig. 4A, Set2FW and 2PRV, expected product size 235 bp). Another set spanned exons 4 and 5 for detection of all isoform transcripts (Fig. 4A, FW and RV, expected product size 160 bp). It should be noted that it was not possible to detect all transcripts due to the small exon sizes and low transcription levels. In the highly methylated HCT116 cells and the untreated SH-SY5Y cells, RT-PCR indicated the silencing of all *ZFP57* isoforms (Fig. 4C, D). Reduced DNA methylation in the DKO- and Aza-treated cell lines allow amplification of exons 4 and 5 including the canonical RefSeq transcript. Transcripts containing exons 1 and 2 were also detectable in these cell lines, indicating the presence of the alternative isoforms (Fig. 4C, D). It is clear from this analysis and our previous work [31] that DNA methylation within the DMR of the alternative upstream promoter controls the transcription of all known *ZFP57* isoforms.

Differences have been reported in the presence or absence of exon 2 on the alternative splice isoforms (compare NM_001109809.4 and NM_ 001366333.2, Fig. 4A). We decided to examine possible spliced variants of *ZFP57* using RNA-Seq in human placenta from publicly available data. Confirmation that some transcripts began at exon 2 in that tissue was obtained from a Shashimi plot (Fig. 4E): these correspond with the original RefSeq transcript extending from exons 2–5. Additionally, variants that originated at the upstream exon 1 and skipped exon 2 were seen, corresponding to NM_001109809.4, which would produce a protein with fewer residues at the amino terminus. Using the primers Set2F-RV (indicated in Fig. 4A), we cloned and sequenced a PCR product spanning exons 1–5 from the Aza-treated neuroblastoma cell line. Sequencing of this transcript, which started at the novel promoter in the DMR, showed the presence of exon 2 and all the downstream exons, corresponding to NM_ 001366333.2, confirming that the 5’ exon can splice into exon 2 to produce a full-length open reading frame (Fig. 4E).

### Analysis of cognitive testing in children at 11 years shows a protective effect of folic acid

During the 11-year period following the FASSTT RCT the developing children were monitored. We examined possible links between cognitive improvements at 11 years and mQTL genotypes for *ZFP57.* Several cognitive results were compared for correlations with the mQTL genotypes and their methylation patterns. We first focussed on data from the WISC-IV clinical assessment tool, where previously collected data including scores for Full Scale IQ, as well as Verbal Comprehension and Processing Speed, where the latter includes the subtests symbol search and cancellation [32].

**Fig. 5:**
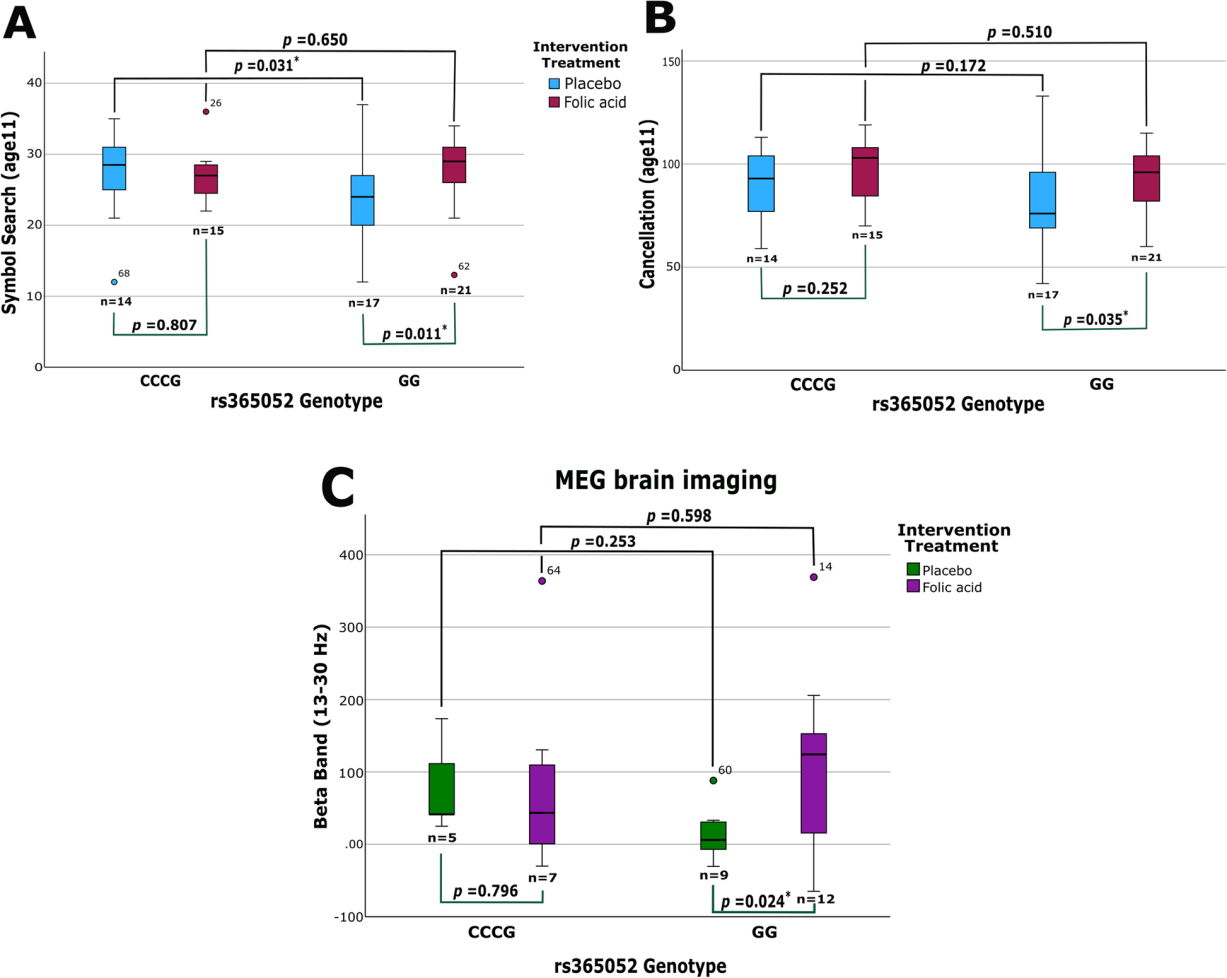
Cognitive and MEG analysis.** A** Cognitive scores for symbol search at each rs365052 genotype, split by intervention group. Scaled scores were used to correct for any small differences in age. Significant *p* value: *p* < 0.05 tested via ANOVA. **B** The same as in **A** but for the other processing speed test, cancellation. Significant *p* value: *p* < 0.05 tested via ANOVA. **C** MEG data for the beta band subgroup (13–30 Hz) showing results for participants between the different rs365052 genotypes, split by intervention group. Significant *p* value: *p* < 0.05 tested via ANCOVA, confounding for sex

Here we examined whether continued FA supplementation during pregnancy may have protective effects on symbol search and cancellation abilities for those children who carry a specific genotype. Due to a lack of C/C genotype samples who also participated in the 11-year cognitive tests, the C/C and C/G groups were combined for this analysis. When comparing intervention groups for these genotypes against symbol search and cancellation scores there were significant differences through ANOVA analysis. In the symbol search category, the CC/CG genotype scored significantly higher than the G/G genotype (*p* = 0.031) in the placebo group (Fig. 5A). The FA group showed no significant difference (*p* = 0.650) between the genotypes. We next compared each genotype separately between the intervention groups. There is no significant difference between the placebo group symbol search score and the FA symbol search scores for the CC/CG genotype (*p* = 0.807). The G/G genotype however, showed significant differences between the FA and placebo groups (*p* = 0.011). These results indicate that G/G individuals performed more poorly than CC/CG individuals in the placebo group, but this effect was not seen in the supplemented group.

For cancellation (Fig. 5B), no significant difference can be seen between the intervention groups for the CC/CG genotype (*p* = 0.252), the placebo group between genotypes (*p* = 0.172) or the FA group between genotypes (*p* = 0.510). However, the G/G group did show significant differences when split by intervention groups (*p* = 0.035), with the placebo group here scoring lower than the FA group, similar to the same investigation in symbol search. The placebo G/G group symbol search and cancellation scores diverge away from the other groups. However, the FA G/G group remained closer to that of the CC/CG in both intervention groups. Furthermore, as a control, we assessed the rs365052 genotypes for both symbol search and cancellation without splitting the data into intervention groups, no significant differences were identified (Additional File 3: Fig. S4). Additional statistical tests were also performed for Full Scale IQ and verbal comprehension as previous analysis has reported differences between placebo and intervention groups [32], but no significant results were discovered.

### Brain imaging analysis supports interactions

We next sought to validate using a second method the differences in cognitive development shown by the WISC-IV test. A subset of the children was successfully recruited for imaging of neuronal activity using a magnetoencephalogram (MEG) while carrying out a language task. This task was chosen as it was considered most analogous to the cognitive processes showing differences in the WISC-IV. Figure 5C shows brain scan data (beta band, 13–30 Hz) from this analysis. The pattern that emerged from the processing speed tests again is evident here. Similar to the analysis conducted for the cognitive assessments, the data produced from MEG brain imaging was split by rs365052 genotypes and intervention groups (Fig. 5C, placebo in green, FA in purple). When comparing the G/G genotype only between the intervention groups, the placebo G/G genotype displayed significantly lower power in the beta band for the language paradigm than the G/G FA group (*p* = 0.024). However, there were no statistically significant differences when the CC/CG group was compared between the genotypes or when the CC/CG genotype was compared to the G/G genotype.

Overall, the children who have a G/G genotype at the rs365052 mQTL and whose mothers were part of the FA-supplemented group of the FASSTT study showed a protective cognitive effect from FA. This group of children had maintained scores in two processing speed tests (symbol search and cancellation) and in MEG analysis (beta band) that was in line with the children who have a CC/CG genotype at this mQTL. The children that have a G/G genotype but were from the mothers who were in the placebo group, did not maintain these scores and show a decreased cognitive ability in these categories.

### Haplotype also predicts methylation and neurocognitive outcomes in the wider population

We have shown above that rs365052 and rs2747429 both show a clear mQTL pattern in our cohort. The analysis showed that in both mQTLs the C/C genotypes are inherited together meaning a blood sample with a rs365052 genotype of C/C will have low levels of methylation in *cis*. In addition, the samples rs2747429 genotype will be C/C as it too tracks with low levels of local methylation. Those that were confirmed as heterozygous for rs365052 (C/G) also had a heterozygous rs2747429 genotype (C/T). Lastly, rs365052 genotypes with the highly methylated homozygous G/G also had a highly methylated homozygous genotype for rs2747429 (T/T).

**Fig. 6:**
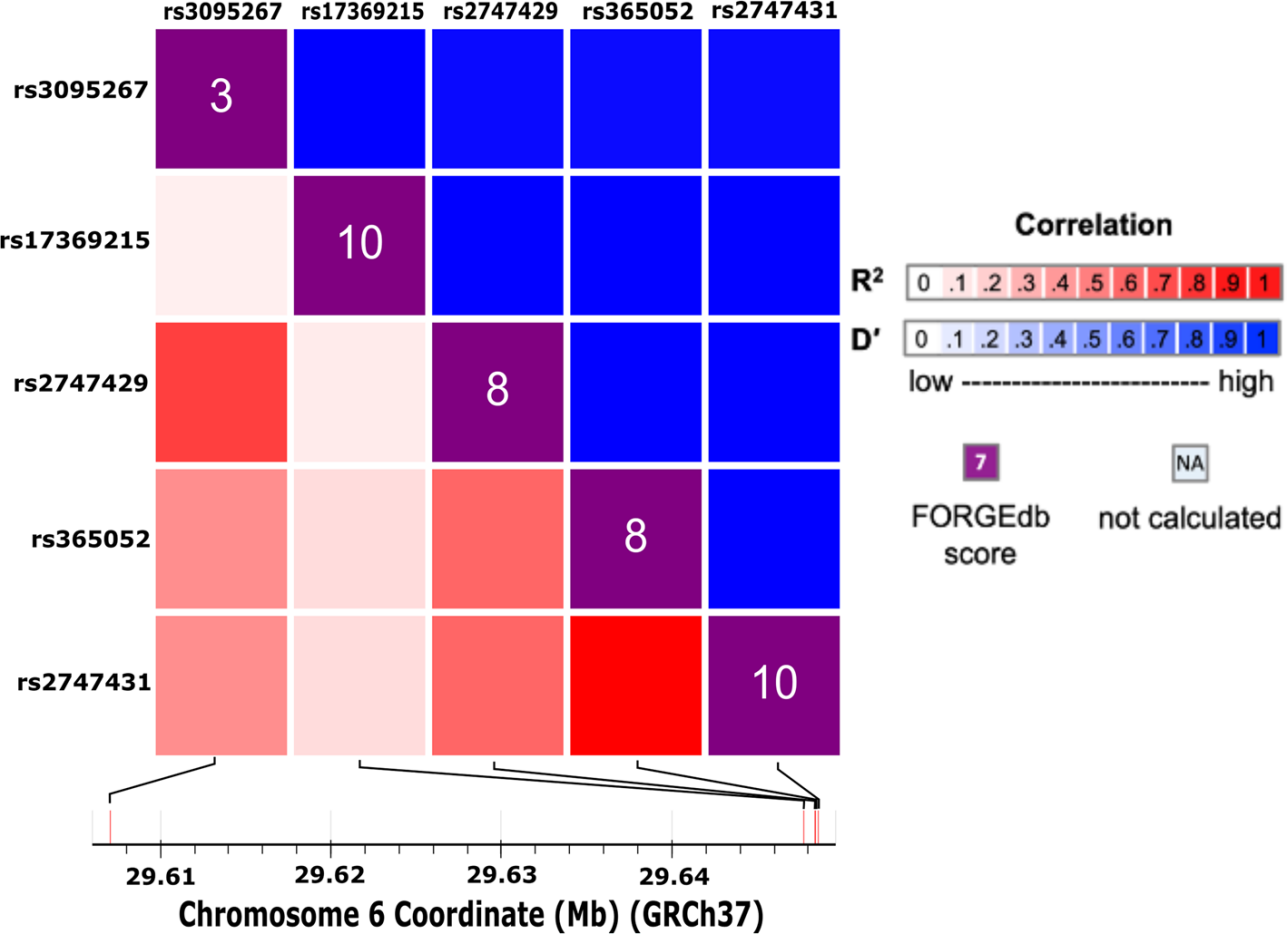
Linkage disequilibrium heatmap*.* Heatmap of data from the 1000 Genomes Project for SNPs in the *ZFP57* promoter region. The bottom left squares represent the *R*.^2^ values (red) and the top right represents the *D*’ (blue) values. The intensity of the colour indicates how close the values are to 1 as seen on the right. A score of 1 is classified as complete linkage disequilibrium while 0 is equilibrium. The diagonal line of squares in the middle is the Functional OR Genomic Element Database (FORGEdb) scores which are out of 10 and is used to predict if genetic variants are likely to be regulatory elements. The closer to 10, the more likely this is. The bottom section shows where each SNP is situated on the gene with chromosome coordinates [53]

To further investigate these findings in the general population, data from the 1000 Genomes Project was used and LD scores calculated (Fig. 6). A heatmap was generated comparing the two mQTLs discussed, along with three additional SNPs in close proximity. The heatmap is used to visualise LD between the four SNPs. The bottom left indicates the *R* squared (*R*^2^) value via different intensities of red. *R* squared is a measure of correlation of alleles and ranges from 0 to 1 (0 represents independent SNPs, 1 represents that one SNP allele perfectly predicts another SNP allele). The top right (blue) indicates the D prime (D’) values for each SNP but were less informative compared to the *R*^2^ values. D prime is an indicator of allelic segregation of SNPs and ranges from 0 to 1 (0 indicates no linkage between SNP alleles, 1 represents a tight linkage of SNP alleles). A key is displayed with the heatmap and provides a value to the corresponding colour intensity. A table was generated with the exact *R*^2^ values for each SNP comparing to the other four (Table 5). The primary SNP in this investigation (rs365052) showed a perfect LD *R*^2^ score of 1.0 with a SNP in close proximity (rs2747431). Moreover, a moderate association can be seen between the two mQTLs rs365052 and rs2747429 (*R*^2^ = 0.598). The mQTL rs365052 also had a slightly weaker but still moderate association with another SNP, rs3095267 (*R*^2^ = 0.444). However, there was only a weak association with the last SNP investigated (rs17369215). This SNP was the second furthest away from rs365052 at a distance of 638 bp downstream, compared to 21 bp downstream (rs2747429) and 166 bp upstream (rs2747431). However, the SNP furthest away was rs3095267, that is located 41,352 bp downstream of rs365052 and is not within the boundaries of the *ZFP57* gene. The secondary mQTL investigated here (rs2747429) had moderate associations with rs365052 and rs2747431 (*R*^2^ = 0.598). Although this too had a very weak correlation with rs17369215 (*R*^2^ = 0.082), it had a strong correlation with rs3095267 (*R*^2^ = 0.751).

**Table 5:** *R* squared (*R*^2^) values for each SNP showing correlations between them

RS_number	rs17369215	rs2747429	rs365052	rs2747431	rs3095267	Association
rs17369215	1.0	0.082	0.137	0.137	0.066	Cognitive performance (MTAG), transient neonatal diabetes mellitus
rs2747429	0.082	1.0	0.598	0.598	0.751	Transient neonatal diabetes mellitus
rs365052	0.137	0.598	1.0	1.0	0.444	Transient neonatal diabetes mellitus
rs2747431	0.137	0.598	1.0	1.0	0.444	Severe COVID-19 or systemic lupus erythematosus (MTAG), transient neonatal diabetes mellitus
rs3095267	0.066	0.751	0.444	0.444	1.0	Migraine without aura

*MTAG* multi-trait analysis of genome-wide association studies (GWAS)

In addition to *R*^2^ scores, each SNP was rated from 1 to 10 using the Functional OR Genomic Element Database (FORGEdb) ranking system. These scores help predict how likely a genetic variant is to be a regulatory variant. The purple boxes on the heatmap (Fig. 6) display the score given to each SNP; the closer the score is to 10, the more likely it is to be a regulatory variant. Both of the mQTLs investigated here (rs365052 and rs2747429) were given scores of 8/10. The other two SNPs from the database were given scores of 10/10. In relation to the primary mQTLs, an 8/10 would indicate that they are both highly likely to be regulatory variants and therefore both have a high probability of affecting gene expression. Furthermore, rs3095267 had a low score of 3/10. This would indicate it is not likely to be a regulatory variant and considering that it is far away from the other SNPs and its closest gene is ~ 6000 bp downstream this score seems reasonable.

## Discussion

We have shown here that a primary regulator of the *ZFP57* gene in humans is DNA methylation. We identified a mQTL variant important in allele-specific DNA methylation and ultimately gene transcription. This mQTL resides within a DMR of the first intron on an alternative *ZFP57* transcript. We have shown that the alternative 5’ promoter of *ZFP57* can be found in neural tissues such as neuroblastoma cells. In addition, suppression of this region can be controlled by changes in DNA methylation at the DMR. We highlighted that this region contains a CTCF site, but further investigation is needed to fully elucidate its complete function. We have demonstrated that there are multiple SNPs in LD, some of which are mQTLs affecting methylation of the region. Using a RCT as a robust design, we have highlighted an association between FA supplementation and a potential cognitive protective mechanism. This is primarily for carriers of a particular genotype at the rs365052 mQTL.

Several studies including our own have already shown that methylation at the *ZFP57* gene is responsive to environmental changes. In particular, we and others have shown that differences can be seen in response to folate status [31, 56]. Work from the Tycko lab has also previously shown that allele-specific methylation of this region of *ZFP57* is haplotype-dependent, suggesting that genetic variants here play an important role in determining methylation, particularly in brain tissue [55]. Allele-specific methylation was also reported in human embryonic stem cells (hESC) for this region [25]. However, neither of these studies looked at a human cohort and much of the mapping was done in cell lines or limited tissue samples. Here we used blood samples from our FASSTT RCT to confirm polymorphism at two mQTLs in this cohort. Our data support the existence of mQTLs at this locus and through examination of mother–child pairs could also exclude an imprinting effect at this locus, since G/G homozygotic mothers showed near 100% methylation rather than the 50% expected if the region was imprinted. By analysing the results in our longitudinal cohort, we also show a clear interaction with nutrition in determining methylation, and by extension transcription, at the *ZFP57* locus, with nutrition having a smaller effect (approx. 5% differences in our cohort) while the mQTLs have a larger, additive effect (close to 50% for a C allele).

The mQTL rs2747429 resides 21 base pairs downstream from our mQTL (rs365052) and has already been recognised as a mQTL in brain and T-cells [55]. We included some analysis of this mQTL as a control for our own. The close proximity of the two mQTLs discussed here would indicate a high probability of linked inheritance; however, our cohort is not large enough on its own to confirm this. We used existing data from the 1000 Genomes Project to calculate the *R*^2^ correlation value to determine LD strength. A British population was selected to best compare to our FASSTT participants from Northern Ireland. This highlighted a moderate level of LD present between the two mQTLs rs365052 and rs2747429, supporting our most recent data of mQTL genotype inheritance. Three other SNPs we examined within the region that had been previously associated with the mQTL rs365052 [53, 54, 57, 58]. A perfect LD score was shown between the mQTL rs365052 and rs2747431 that also resides in the DMR of *ZFP57*. This SNP has been previously associated with TNDM, severe COVID-19 and systemic lupus erythematosus [58]. The SNP rs3095267 showed a moderate LD score with our mQTL rs365052 and a strong score with mQTL rs2747429 and has been previously associated with migraines [59]. The mQTL rs365052 also showed evidence of linkage to the SNP rs17369215, which is associated with cognitive performance as well as TNDM [57]. Although the score was not high, it has been suggested that any score over 0.1 be classified as in LD [53]. This SNP rs17369215 resides outside of the CTCF insulator region marked by ChromHMM in ESCs but lies within the larger CTCF binding site illustrated by ENCODE3 transcription factor binding ChIP-Seq data on 130 cell types on UCSC. Therefore, as well as linking this region to TNDM and the associated multi-locus imprinting disorder (MLID), in which *ZFP57* has been implicated, these linkage relationships indicate a causative role for this region in certain neurophysiological phenotypes including cognition [28, 30, 60].

In terms of evolutionary conservation, it is reasonably well-established that *ZFP57* plays an important role in the regulation of imprinted genes in both mouse and human tissue [17, 25, 29, 61–63]. The translated KRAB-ZF protein has recently been shown to have a more diverse binding ability than previously thought, reaching beyond just imprinted genes to include factors such as transposable elements [63]. Due to its wide binding ability and involvement in different genetic and epigenetic mechanisms alongside its location within the MHC, regulation of this unique gene is of great importance [24, 64]. Moreover, there is growing evidence of alternative *ZFP57* isoforms in multiple tissues [25]. Two of these newer isoforms start approximately 3 kb upstream from the canonical TSS with the first exon overlapping that of our DMR. The presence or absence of the second exon in the transcript is what sets the two newer isoforms apart from each other. This second exon represents the start of the previously characterized RefSeq transcript [25]. We have confirmed here the presence of the alternative transcript upstream of the original RefSeq *ZFP57* transcript using a neuroblastoma cell line. With the aim to uncover the regulatory abilities of this mQTL-associated alternative promoter on the *ZFP57* isoforms, we decided to investigate if methylation of this influential DMR could alter the expression of the newer *ZFP57* isoforms. We deemed it most appropriate to use cell line pairs that had only one factor different between them, namely the presence of absence of DNA methylation. We used a commercially derived cell line (HCT116) and its DKO counterpart alongside the neuroblastoma cell line (SH-SY5Y) and its Aza-treated counterpart. Our previous analysis of these two systems highlighted altered gene expression of *ZFP57* without isolation of the alternative isoforms [31]. Our most recent data demonstrates how altering DNA methylation alone can facilitate an upregulation of all *ZFP57* isoforms. This reinforces the obvious role DNA methylation plays within the *ZFP57* alternative promoter and its relationship with all transcripts. Furthermore, the significant role that the mQTL (rs365052) plays in driving *cis* methylation can lead to altered expression of the *ZFP57* gene.

It is well established that CTCF can regulate gene expression and is known for its insulator properties, blocking enhancer–promoter interactions for targeted genes [65]. This protein is a ZF transcription factor highly associated with eukaryotes [66, 67]. Publicly available ChIP-seq data from the ENCODE project by multiple labs have localised a CTCF binding site to the insulator region we are investigating in which the mQTLs (rs365052 and rs2747429) reside [68]. However, CTCF has also been shown to interact with other proteins, such as cohesin to modify chromatin through formation of loops to enable interaction between groups of genes in *trans* [69]. It has been shown that CTCF cannot bind methylated sequences and therefore can be disrupted by our informative mQTLs and folate [70]. This disruption can occur directly from a common SNP variant present within the binding site, preventing CTCF’s binding ability [71, 72]. It may also arise through alterations in DNA methylation as a result of the mQTLs within the *ZFP57* alternative promoter. Moreover, multiple CTCF binding sites as well as enhancer regions are shown around the wider *ZFP57* region. It would be of value to identify if these CTCF sites have insulator effects or form loops for regulation of genes in different loci.

The role of *ZFP57* post fertilisation and implantation is yet to be fully portrayed. Further investigations are needed to determine if this gene contributes to regulatory processes in somatic tissues. It can be seen however that *ZFP57* shows significant expression in several adult tissues including the heart, thymus, kidney and brain [24]. Moreover, expanding upon the association between *ZFP57* and transposable elements in human cells could be a worthwhile project for the future [63]. A further area of exploration could be into the evolutionary timeline of these *ZFP57* isoforms. Determining if the longer transcripts developed after the shorter canonical transcript may give valuable insight to their functions. In addition to this, effects beyond imprinted genes to transposable elements or specific non-imprinted regions [63], particularly in the brain should be examined [73]. An important question is whether the results we show here can be extended to brain tissue. In this context, it is important to note that the DMR we show to be responsive to FA and haplotype in cord blood and blood from 11-year-olds also showed allele-specific methylation driven by haplotype in brain tissues in a previous study [55]. This strongly supports a role for the mQTL we examined here in determining methylation levels and hence *ZFP57* transcriptional activity in brain tissues. It is therefore not unreasonable to suppose that FA will also influence methylation and transcription in the brain tissues.

A final discussion involves the action of FA on this region and its association with cognitive development in early life. There is abundant evidence linking FA intake and protection in the first trimester of pregnancy against NTDs such as spina bifida and anencephaly [74]. The FASSTT Offspring Trial showed that continued FA supplementation in the second and third trimesters of pregnancy prevented the reduction of serum folate often seen in pregnancy while increasing maternal and cord blood RBC folate concentrations [41]. This has led to many interesting insights into FA’s role in epigenetic, cognitive and psychosocial development [31–33, 40, 75, 76]. Here we have further explored all three of these areas and have discovered for the first time, a possible protective biological mechanism associated with cognitive processing speed tests and the *ZFP57* mQTL (rs365052). As shown previously, FA is linked to improved cognitive abilities up to 11 years of age [32]. We have highlighted here that an inverse effect which does not directly increase the cognitive abilities of individuals with a particular mQTL variant but instead it may help in maintaining normal development. Participants of the RCT with a G/G genotype in the placebo group showed significantly poorer performance in the Processing Speed tests symbol search and cancellation. However, participants that had the G/G genotype and were in the FA-supplemented group maintained a higher score that aligned with the other genotypes in the study. This same pattern is shown in a subgroup of MEG analysis (beta band) where the placebo G/G group showed significantly lower power than the FA-supplemented G/G group. Interestingly, beta band power has been associated with sensorimotor processing. Increases in beta band power can be seen when physical movement is successfully cancelled, while planning movements or executing movements decreases beta power in the brain [77]. This suggests these children may have better action control and are possibly more focused on the intellectual task in front of them instead of getting distracted. Moreover, a study conducted on children with ADHD used neurofeedback training for theta and beta bands to investigate learning curves and behavioural changes. During this study, the theta band power remained the same while the beta band power increased linearly [78]. Theta band power has been negatively associated to alertness, while beta has been positively associated to attention [79]. We can link all of these complex mechanisms together but deciphering at which stage FA is involved would require further analysis. It is possible that FA acts as a modifier to aid in adjusting the extreme DNA methylation differences attributed to the mQTL genotypes. This may help to regulate the DNA methylation effects as FA is a limiting factor in the one carbon metabolism cycle that is responsible for DNA methylation and synthesis [80].

One of the strengths of the study is the randomised control design, which enables demonstration of a causative link. There is also the use of independent methods to assess cognitive development (WISC-IV, MEG), together with external validation of the association of the mQTL rs365052 at this locus with neurocognitive phenotypes in larger human GWAS cohorts. The consistency in both methylation and cognitive differences between placebo and folic acid groups in different studies and over time for this cohort is also noteworthy [31–33, 40, 49]. One of the main limitations for this study is the relatively small sample size in the RCT cohort, which limits its power to detect differences in some components of the WISC-IV test. There is no comparable RCT cohort looking at cognitive outcomes in children following folic acid supplementation in later pregnancy to which it can be properly compared, although there is some support for improvements in language development and cognitive outcomes from observational cohorts [81–84]. It is also interesting in this context that additional studies have shown effects at the locus in patients suffering from neurocognitive disabilities, including post-traumatic stress disorder [85] and schizophrenia spectrum disorder [64]. It would be valuable to investigate on a larger scale the potential interactions between folate status, haplotype and neurocognitive outcomes in adult cohorts as well as a multi-omics approach to investigate potential effects that occur after gene transcription.

## Conclusions

Despite the limitations discussed, we have shown here that the promoter region of the *ZFP57* gene is controlled by the newly identified mQTL that regulates allele-specific methylation patterns. We showed here that DNA methylation of the promoter silences the alternative isoforms of *ZFP57*. We have also highlighted the presence of additional SNPs/mQTLs in LD possibly collaborating to influence the methylation and expression of *ZFP57*. We have suggested that *ZFP57*’s expression may also be regulated by the transcription factor CTCF located within the DMR overlapping the mQTLs. In addition, we show for the first time a potential interaction between these mQTLs and continued FA supplementation during pregnancy and that this tracks with positive cognitive outcomes in early life. Together, this study sheds light on the regulation of this interesting and epigenetically sensitive gene, as well as providing insights into the potential biological mechanism explaining the beneficial effect of maternal FA during pregnancy on the epigenome and neurodevelopment in the child.

## Supplementary Information

Additional file 1: Additional materials. Supplementary details in relation to investigation overview, primers used and EPIC array probes. Figure S1. Overview showing the analysis of samples from the FASSTT Offspring trial in relation to regulation of *ZFP57*. Table S1. Primer sequences used in this study. Table S2. *ZFP57* EPIC array CpG sites for UCSC human genome 19.

Additional file 2: Methylation and genotypes. Supplementary details in relation to changes in methylation between different tissue timepoints and genotypes of investigated SNPs. Table S3. rs365052 genotypes in the FASSTT participants. Table S4. rs2747429 mQTL genotypes for the FASSTT participants. Table S5. Average methylation levels relevant to the rs2747429 SNP. Figure S2. Boxplot of the rs2747429 SNP methylation in the *ZFP57* alternative promoter region. Table S6. DNA methylation correlations between cord blood and blood at age 11 for the 23 overlapping participants. Figure S3. Tissue methylation comparison of cord blood and blood at 11 years

Additional file 3: Additional cognitive analysis. Supplementary details in relation to cognitive assessments for the rs365052 genotype. Figure S4. Cognitive scores for symbol search and cancellation at each rs365052 genotype

Additional file 4: Original, uncropped gel images. Raw gel images for Figure 4 in the main text. Figure S5. Raw gel images corresponding to Figure 4C and 4D

## Data Availability

Data available upon request.

## Abbreviations

5-aza-dC: 5′Aza-2-deoxycytidine
AQ: Allelic quantification
BMI: Body mass index
cDNA: Complementary DNA
CpG: Cytosine phosphate guanine
CTCF: CCCTC-binding factor
DKO: Double knockout
DMEM: Dulbecco`s modification of Eagle’s medium
DMR: Differentially methylated region
DNAm: DNA methylation
DNMT: DNA methyltransferase
EPIC 850 k Array: Infinium methylation EPIC beadchip array
ESCs: Embryonic stem cells
FPC: Failed primer combinations
FA: Folic acid
FASSTT: Folic Acid Supplementation during the Second and Third Trimester
FBS: Foetal bovine serum
FORGEdb: Functional OR Genomic Element Database
GW: Gestational week
GWAS: Genome-wide association studies
hESC: Human embryonic stem cell
ICR: Imprint control region
Kb: Kilobase
KRAB: Krüppel-associated box
KRAB-ZF: KRAB zinc finger
LD: Linkage disequilibrium
MEG: Magnetoencephalography
MHC: Major histocompatibility complex
MLID: Multi-locus imprinting disorder
mQTL: Methylation quantitative trait locus
NIFBM: Northern Ireland Functional Brain Mapping
NTD: Neural tube defects
RBC: Red blood cell
RCT: Randomised controlled trial
RNA-seq: RNA sequencing
RT: Reverse transcriptase
SNP: Single-nucleotide polymorphism
TNDM: Transient neonatal diabetes mellitus
TSS: Transcriptional start site
UCSC: University of California, Santa Cruz
UV: Ultraviolet
WISC-IV: Wechsler Intelligence Scale for Children fourth edition
WT: Wild type
*ZFP57*: Zinc finger protein 57
