# Supplementary material for: Cognitive benefits of folic acid supplementation during pregnancy track with epigenetic changes at an imprint regulator
Source: BMC Med. 2024 Dec 16;22:579. doi: 10.1186/s12916-024-03804-2 (PMC11650848; doi:10.1186/s12916-024-03804-2)
Supplement: Supplementary file 1 — Additional file 1: Additional materials. Supplementary details in relation to investigation overview, primers used and EPIC array probes. Figure S1. Overview showing the analysis of samples from the FASSTT Offspring trial in relation to regulation of ZFP57. Table S1. Primer sequences used in this study. Table S2. ZFP57 EPIC array CpG sites for UCSC human genome 19. [file 12916_2024_3804_MOESM1_ESM.docx]

**ADDITIONAL FILE 1**

**Additional materials.** Supplementary details including investigation overview or graphical abstract, primers used and EPIC array probes.

**Figure S1.** Overview showing the analysis of samples from the FASSTT Offspring trial in relation to regulation of *ZFP57*.

**Table S1.** Primer sequences used in this study.

**Table S2.** *ZFP57* EPIC array CpG sites for UCSC human genome 19.


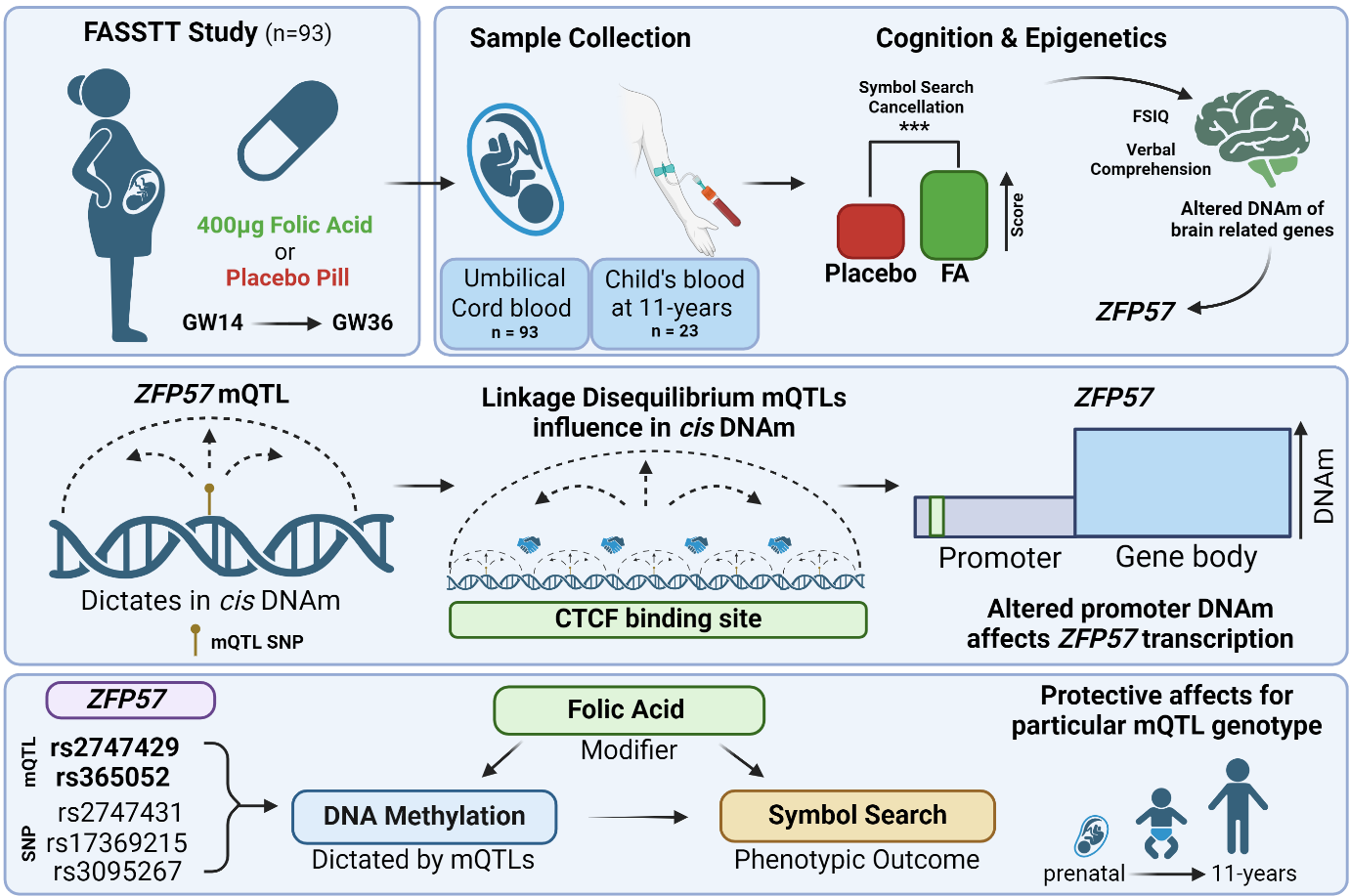


***Figure S1. Overview showing the analysis of samples from the FASSTT Offspring trial in relation to regulation of ZFP57.*** The figure is split into three sections. **Top panel)** depicts the FASSTT trial showing the number of cord blood samples on the left (n=93) and the blood collection timepoints and cognitive test outcomes on the right. **Middle panel)** Shows the DMR promoter on the *ZFP57* gene and how mQTLs work in linkage disequilibrium within the region. **Bottom panel)** Shows the SNPs and mQTLs assessed in this analysis, how DNA methylation, folic acid and the cognitive analysis interlink and how long this effect can be seen in children. Abbreviations: GW, gestational week; FA, Folic Acid; mQTL, methylation Quantitative Trait Locus; DNAm, DNA methylation; SNP, Single Nucleotide Polymorphism; FASSTT, Folic Acid Supplementation in Second and Third Trimester.

**Table S1.** Primer sequences used in this study

| Application | Gene | Primer | Oligo sequence (5`-3`) |
| --- | --- | --- | --- |
| CpG pyroassay | *ZFP57* | FW | GGGATTTTTTTTAGTTATTGTTTTGTAT |
|  |  | RV- 5`Btn | ACTAACAAACCCCTACTTTACCAAAC |
|  |  | Seq | ATTGTTTTGTATTTATTTATTAGA |
| SNP pyroassay | *ZFP57* | FW1 | ACCCTTTCCAGCCATTGC |
|  |  | RV1-5`Btn | GCTAACAAGCGCCTGCTTT |
|  |  | Seq1 | CGCTCCTGGCCCTGT |
|  |  | FW2-5`Btn | CCCTGTCCTCCGCGCTTA |
|  |  | RV2 | GGCTGGGGTTCCGGATCT |
|  |  | Seq2 | GTTCCGGATCTGGGC |
| Clonal analysis | *ZFP57* | FW | GGGATTTTTTTTAGTTATTGTTTTGTAT |
|  |  | RV | ACTAACAAACCCCTACTTTACCAAAC |
| Sequencing of vectors | pJET1.2_vector | Seq | CGACTCACTATAGGGAGAGCGGC |
| RT PCR and RT-qPCR | *ZFP57* | Set2FW | CCCTAAATCGCGGTCCACTG |
|  |  | Set3RV | GCAGGCCTTCTCTCTTAGGC |
|  |  | FW | CCCAAACACAGAAGGCCTTT |
|  |  | RV | GGTCCTGTCCATAGTCCCAG |
|  |  | 2PRV | GGTTTGATGTGGCTTCCTGT |
|  | *HPRT* | FW | AGCCCTGGCGTCGTGATTAGT |
|  |  | RV | CCCGTTGAGCACACAGAGGCCTA |

5`Btn (Biotin tag at the 5` end of the DNA sequence), Seq (sequencing primer)

**Table S2.** *ZFP57* EPIC array CpG sites for UCSC human genome 19.

| cgid | Chromosome | Start |
| --- | --- | --- |
| cg07134666 | chr6 | 29648400 |
| cg00588198 | chr6 | 29648452 |
| cg06032337 | chr6 | 29648468 |
| cg16885113 | chr6 | 29648507 |
| cg20228636 | chr6 | 29648525 |
| cg11383134 | chr6 | 29648590 |
| cg03198009 | chr6 | 29648604 |
| cg03449857 | chr6 | 29648623 |
| cg15570656 | chr6 | 29648628 |
| cg02157626 | chr6 | 29648736 |
| cg13835168 | chr6 | 29648756 |
| cg08041448 | chr6 | 29648901 |
| cg24100841 | chr6 | 29649024 |
| cg19636627 | chr6 | 29649084 |
| cg05863862 | chr6 | 29649807 |
