# Supplementary material for: Cognitive benefits of folic acid supplementation during pregnancy track with epigenetic changes at an imprint regulator
Source: BMC Med. 2024 Dec 16;22:579. doi: 10.1186/s12916-024-03804-2 (PMC11650848; doi:10.1186/s12916-024-03804-2)
Supplement: Supplementary file 2 — Additional file 2: Methylation and genotypes. Supplementary details in relation to changes in methylation between different tissue timepoints and genotypes of investigated SNPs. Table S3. rs365052 genotypes in the FASSTT participants. Table S4. rs2747429 mQTL genotypes for the FASSTT participants. Table S5. Average methylation levels relevant to the rs2747429 SNP. Figure S2. Boxplot of the rs2747429 SNP methylation in the ZFP57 alternative promoter region. Table S6. DNA methylation correlations between cord blood and blood at age 11 for the 23 overlapping participants. Figure S3. Tissue methylation comparison of cord blood and blood at 11 years [file 12916_2024_3804_MOESM2_ESM.docx]

**ADDITIONAL FILE 2**

**Methylation and genotypes.** Supplementary details in relation to the genotypes of investigated SNPs, associated methylation levels, and correlations between tissues and timepoints.

**Table S3.** rs365052 genotypes in the FASSTT participants.

**Table S4.** rs2747429 mQTL genotypes for the FASSTT participants.

**Table S5.** Average methylation levels relevant to the rs2747429 SNP.

**Figure S2.** Boxplot of the rs2747429 SNP methylation in the *ZFP57* alternative promoter region.

**Table S6.** DNA methylation correlations between cord blood and blood at age 11 for the 23 overlapping participants.

**Figure S3.** Tissue methylation comparison of cord blood and blood at 11 years.

**Table S3.** rs365052 genotypes in the FASSTT participants

|  | Placebo (n=51) | Folic acid (n=40) |
| --- | --- | --- |
| Overall *p* value 0.524 | N | N |
| rs365052 SNP Genotype, CC | 6 | 2 |
| rs365052 SNP Genotype, CG | 20 | 15 |
| rs365052 SNP Genotype, GG | 25 | 23 |

Statistical comparisons by Fisher’s Exact test. Genotyping performed by AQ pyrosequencing. N, number of samples; **p*< 0.05.

**Table S4.** rs2747429 mQTL genotypes for the FASSTT participants

| Overall *p* value 0.819 | Placebo (n=9) | Folic acid (n=13) |
| --- | --- | --- |
|  | N | N |
| rs2747429 SNP Genotype, CC | 1 | 2 |
| rs2747429 SNP Genotype, CT | 1 | 3 |
| rs2747429 SNP Genotype, TT | 7 | 8 |

Statistical comparisons by Fisher’s Exact test. Genotyping performed by AQ pyrosequencing. N, number of samples; **p*< 0.05.

**Table S5.** Average methylation levels relevant to the rs2747429 SNP.

| rs2747429 variants | Av. Meth. | SD | Min | Max | N | *p* value |
| --- | --- | --- | --- | --- | --- | --- |
| CC | 18.99 | 6.88 | 14.69 | 26.93 | 3 | *CT = 9.95E-4* |
|  |  |  |  |  |  | *TT = 9.79E-8* |
| CT | 48.28 | 4.08 | 42.66 | 52.15 | 4 |  |
| TT | 72.24 | 8.60 | 59.49 | 84.31 | 15 | *CC = 9.79E-8* |
|  |  |  |  |  |  | *CT = 3.63E-4* |

Av. Meth., average methylation between 6 CpG sites measured by pyrosequencing; SD, standard deviation; min, minimum; max, maximum; N, number of samples; significance indicated by *p*<0.05 and is measured by ANOVA between the genotypes.


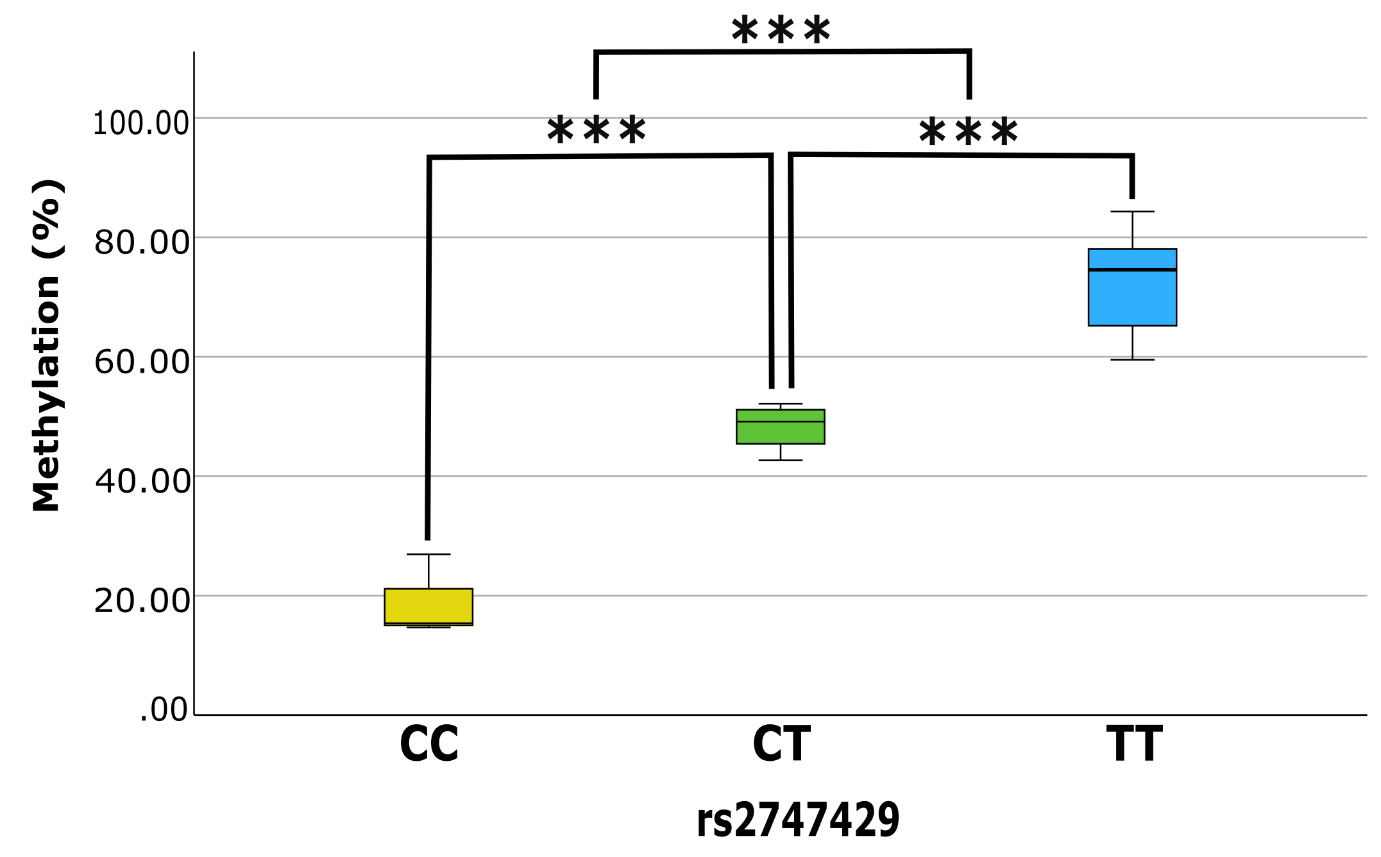


***Figure S2.*** **Boxplot of the rs2747429 SNP methylation in the *ZFP57* alternative promoter region**. Significant methylation differences are shown between different genotype groups in cord blood. Genotype analysis performed by AQ pyrosequencing. Statistically significant values are marked as: *** indicates *p*<0.001, conducted by ANOVA.

**Table S6.** DNA methylation correlations between cord blood and blood at age 11 for the 23 overlapping participants.

| **Tissue type** | **N** | **Av. Meth.** | **Standard Deviation** | **Cord Blood** | **11-year blood** | **Spearman (Non-parametric)** |
| --- | --- | --- | --- | --- | --- | --- |
| Cord Blood | 23 | 65.91 | 16.71 | 1 | 0.423 | Correlation Coefficient |
|  |  |  |  | - | 0.044* | Sig. (2-tailed) |
| 11-year blood | 23 | 55.41 | 8.98 | 0.423 | 1 | Correlation Coefficient |
|  |  |  |  | 0.044* | - | Sig. (2-tailed) |

Correlations conducted through non-parametric Spearman’s rank correlation coefficient. Av. Meth., average methylation between 6 CpG sites measured by pyrosequencing; statistically significant results displayed by asterisk: * indicates *p*<0.05.


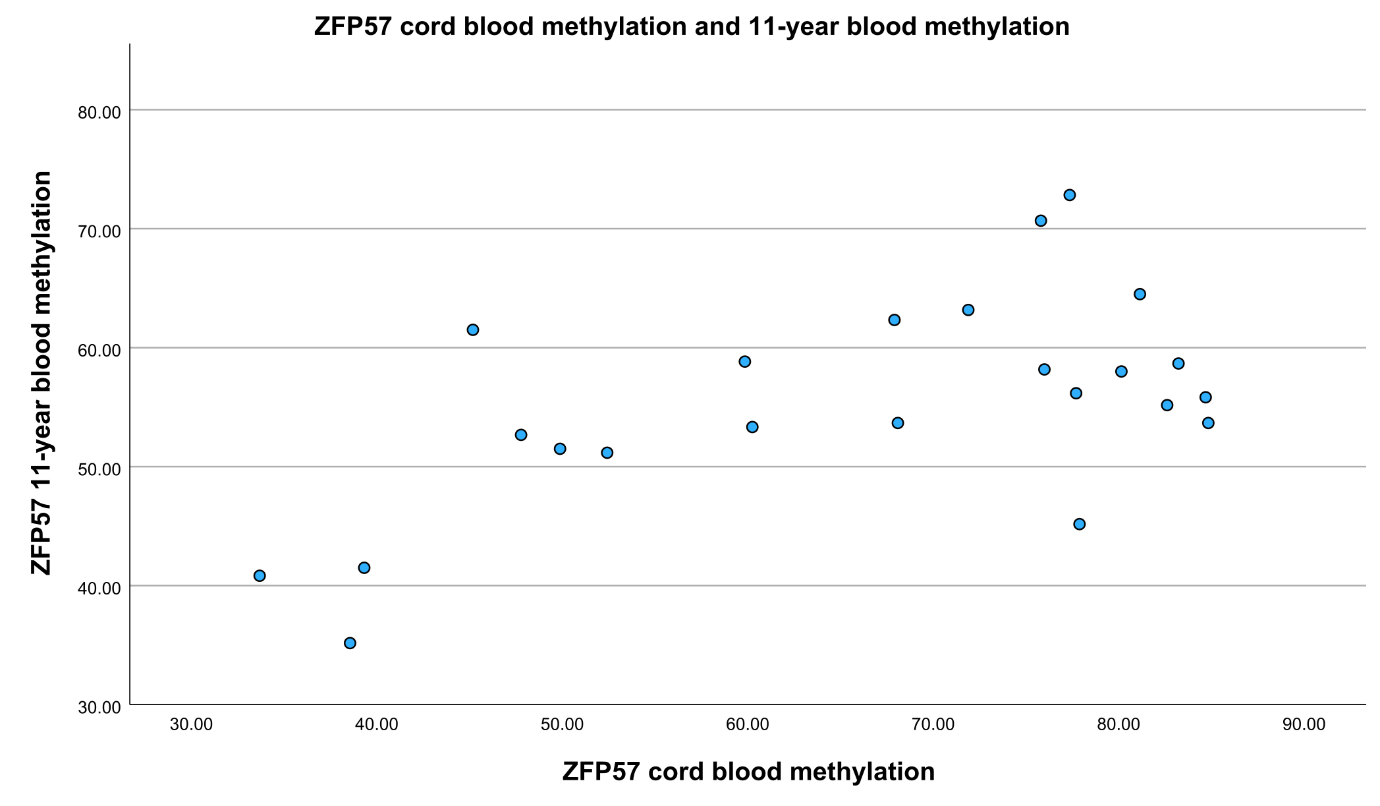
 ***Figure S3. Tissue methylation comparison of cord blood and blood at 11 years.*** Correlation plot of the 23 overlapping participant samples for cord blood and blood from 11-year-olds. Average methylation at the 6 CpG sites at the *ZFP57* alternative promoter region is displayed for each tissue type.
