# Supplementary material for: Cognitive benefits of folic acid supplementation during pregnancy track with epigenetic changes at an imprint regulator
Source: BMC Med. 2024 Dec 16;22:579. doi: 10.1186/s12916-024-03804-2 (PMC11650848; doi:10.1186/s12916-024-03804-2)
Supplement: Supplementary file 3 — Additional file 3: Additional cognitive analysis. Supplementary details in relation to cognitive assessments for the rs365052 genotype. Figure S4. Cognitive scores for symbol search and cancellation at each rs365052 genotype [file 12916_2024_3804_MOESM3_ESM.docx]

**ADDITIONAL FILE 3**

**Additional cognitive analysis.** Supplementary details in relation to cognitive assessments for the rs365052 genotype.

**Figure S4.** Cognitive scores for symbol search and cancellation at each rs365052 genotype.


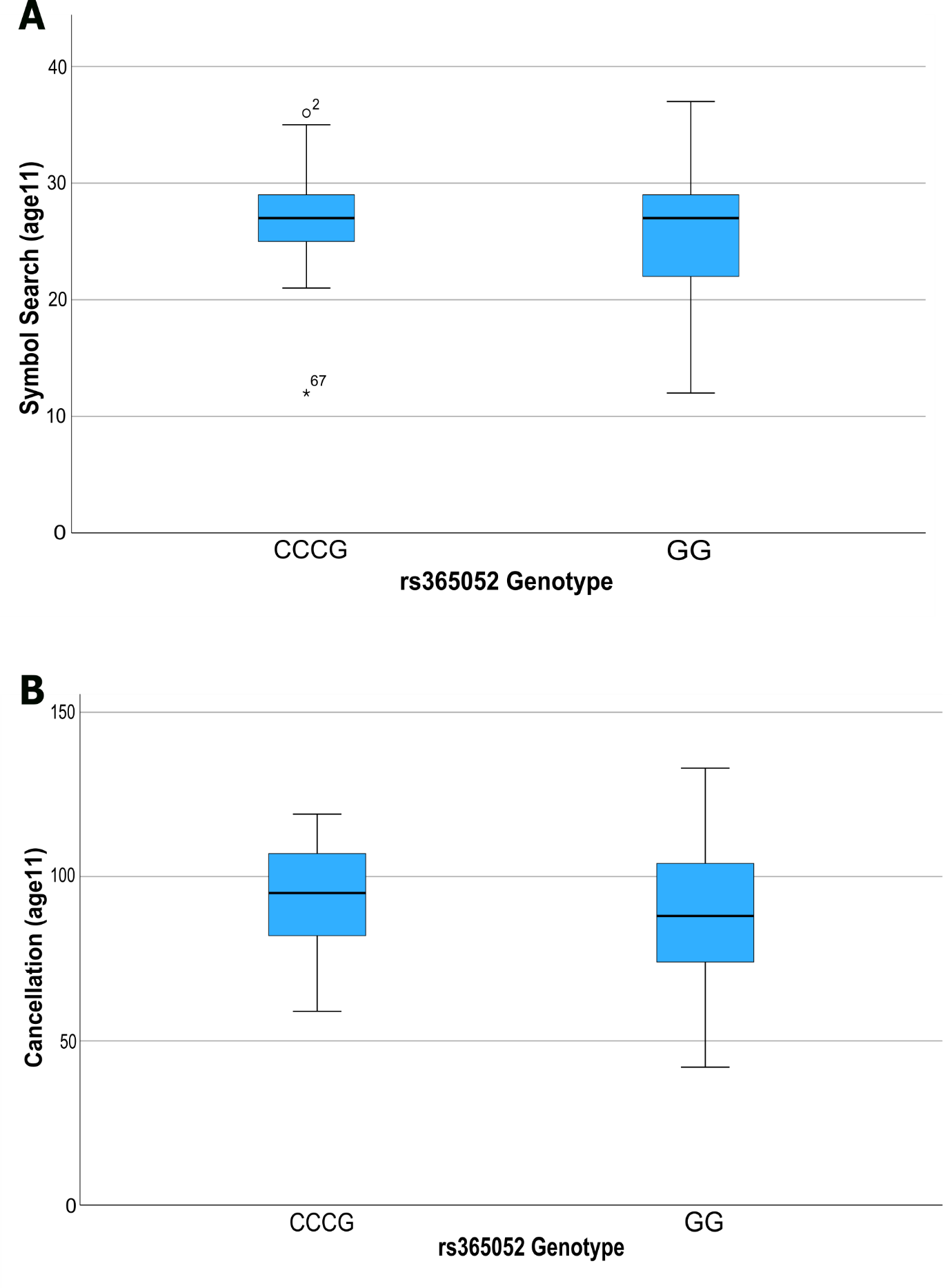
***Figure S4.* A)** Cognitive scores for symbol search at each rs365052 genotype. Non-significant 2-sided *p* value from independent samples t-test = 0.227. **B)** Cognitive scores for cancellation at each rs365052 genotype. Non-significant 2-sided *p* value from independent samples t-test = 0.227.
