# Supplementary material for: Cognitive benefits of folic acid supplementation during pregnancy track with epigenetic changes at an imprint regulator
Source: BMC Med. 2024 Dec 16;22:579. doi: 10.1186/s12916-024-03804-2 (PMC11650848; doi:10.1186/s12916-024-03804-2)
Supplement: Supplementary file 4 — Additional file 4: Original, uncropped gel images. Raw gel images for Figure 4 in the main text. Figure S5. Raw gel images corresponding to Figure 4C and 4D [file 12916_2024_3804_MOESM4_ESM.docx]

**ADDITIONAL FILE 4**

**Original, uncropped gel images.** Raw gel images for Figure 4 in the main text.

**Figure S5.** Raw gel images corresponding to Figure 4C and 4D.


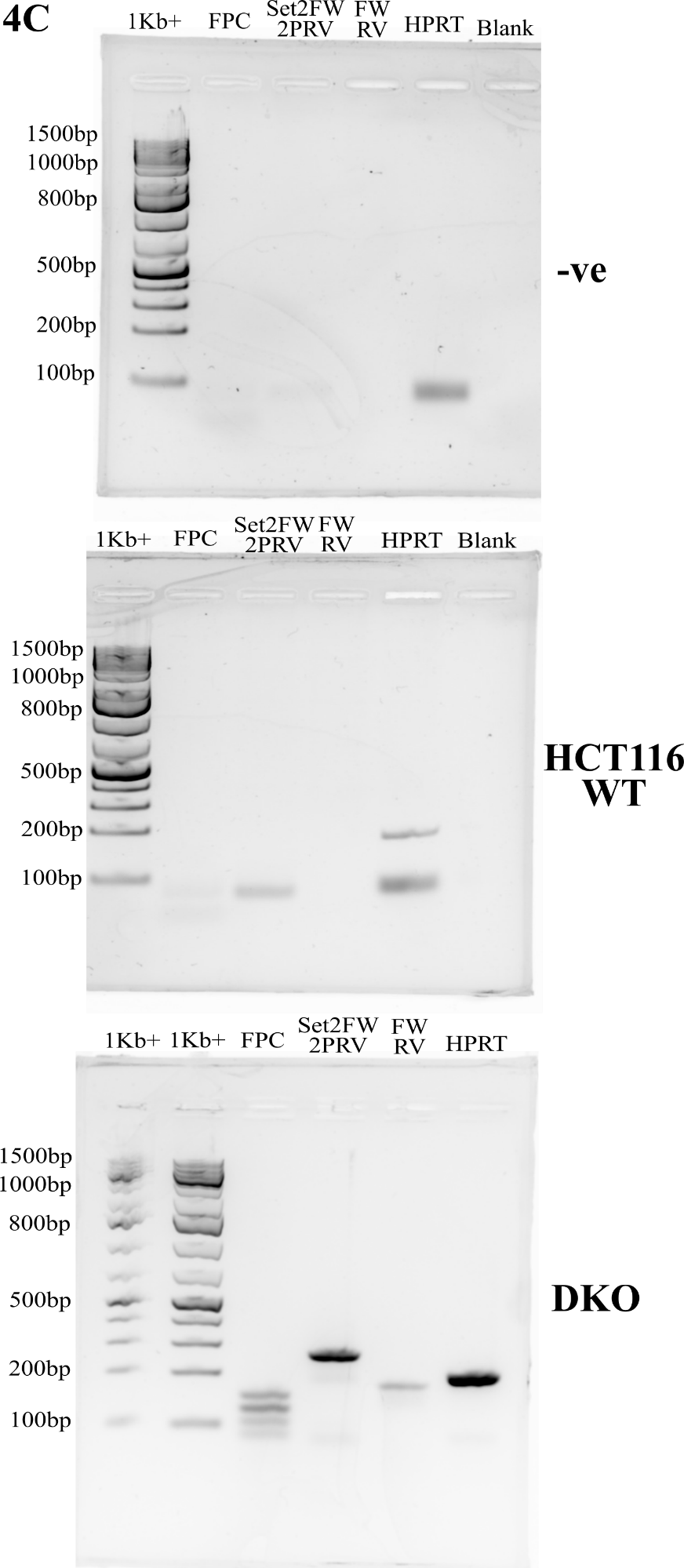

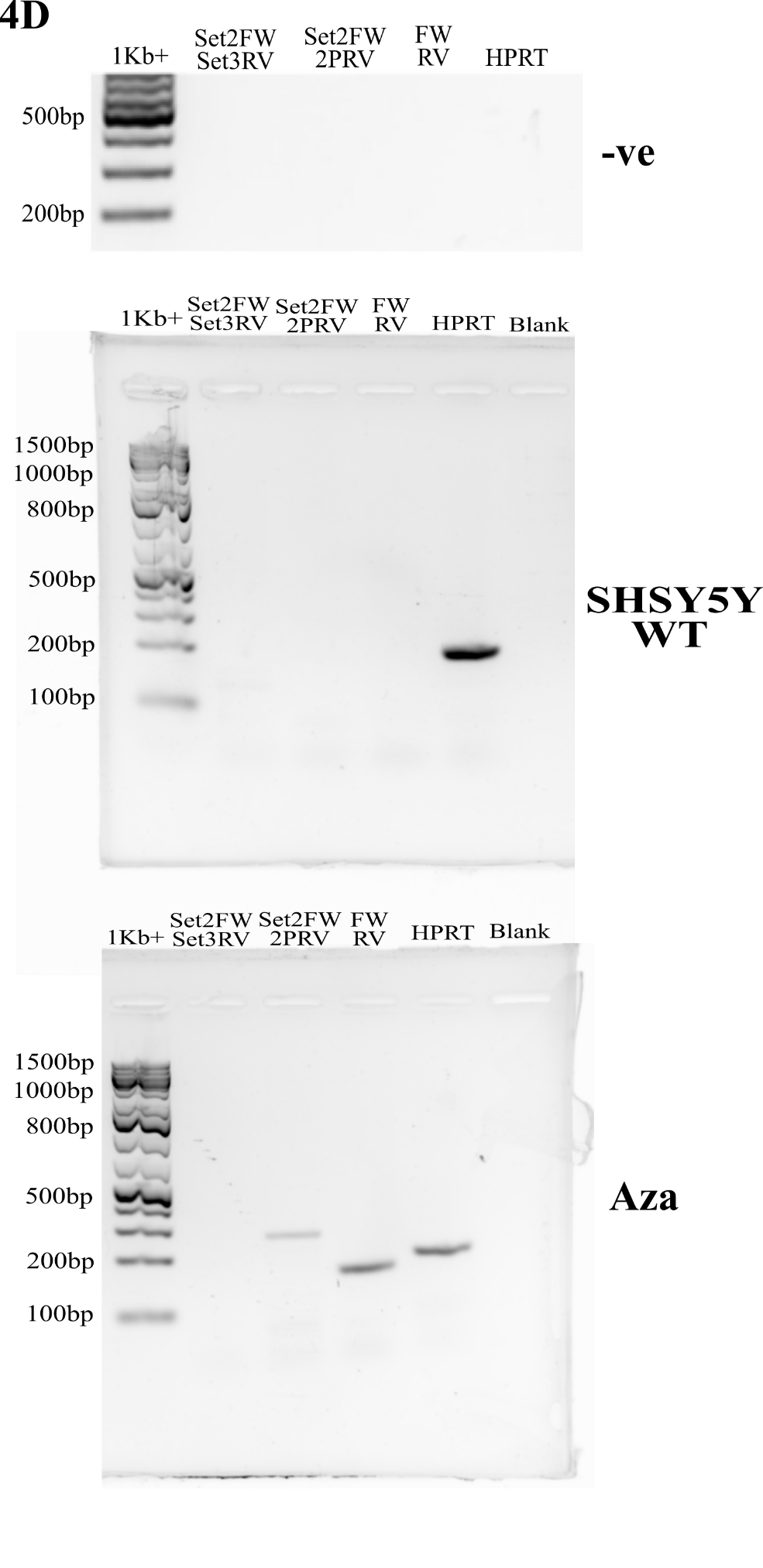


***Figure S5. Raw gel images for HCT116 and SH-SY5Y cell lines, corresponding to Figure 4C and Figure 4D.* 4C)** RT-PCR showing upregulation of the individual exon junctions using the primers indicated in Figure 4A, in human colorectal cancer cells, **4D)** in the neuroblastoma cell line SH-SY5Y. SH-SH5Y -ve full gels not found. DKO, Double Knockout; WT, Wild Type; -ve, negative; FPC, Failed primer combinations.
